# *MAFG‐AS1* promotes tumor progression via regulation of the HuR/PTBP1 axis in bladder urothelial carcinoma

**DOI:** 10.1002/ctm2.241

**Published:** 2020-12-16

**Authors:** Mengqing Xiao, Jianye Liu, Liang Xiang, Kai Zhao, Dong He, Qinghai Zeng, Qun Zhang, Dan Xie, Minhua Deng, Yuxing Zhu, Yeyu Zhang, Yan Liu, Hao Bo, Xiaoming Liu, Xingyu Chen, Lian Gong, Ying Bao, Yi Hu, Yaxin Cheng, Liping Deng, Rongrong Zhu, Xiaowei Xing, Ming Zhou, Wei Xiong, Yanhong Zhou, Jianda Zhou, Xiaohui Li, Ke Cao

**Affiliations:** ^1^ Department of Oncology Third Xiangya Hospital of Central South University Changsha China; ^2^ Department of Urology Third Xiangya Hospital of Central South University Changsha China; ^3^ Department of Hematology Third Xiangya Hospital of Central South University Changsha China; ^4^ Department of Respiratory The Second People's Hospital of Hunan Province Changsha China; ^5^ Department of Dermatology Third Xiangya Hospital of Central South University Changsha China; ^6^ Department of Radiotherapy The First Affiliated Hospital of Sun Yat‐sen University Guangzhou China; ^7^ Department of Pathology Sun Yat‐sen University Cancer Center Guangzhou China; ^8^ Department of Urology Sun Yat‐sen University Cancer Center Guangzhou China; ^9^ Department of Plastic Surgery Third Xiangya Hospital of Central South University Changsha China; ^10^ Institute of Reproductive and Stem Cell Engineering Central South University Changsha China; ^11^ Department of Gastroenterology Third Xiangya Hospital of Central South University Changsha China; ^12^ Center for Medical Experiments Third Xiangya Hospital of Central South University Changsha China; ^13^ Cancer Research Institute and Key Laboratory of Carcinogenesis of the Chinese, Ministry of Health Central South University Changsha China; ^14^ Hunan Key Laboratory for Bioanalysis of Complex Matrix Samples Changsha China; ^15^ Department of Pharmaceutical Chemistry, School of Pharmaceutical Sciences Central South University Changsha China

**Keywords:** bladder urothelial carcinoma, HuR, *MAFG‐AS1*, PTBP1

## Abstract

Long noncoding RNAs (lncRNAs) play a crucial role in progression of bladder urothelial carcinoma (BUC). However, the molecular mechanisms behind this role have not been elucidated yet. Here, we found that the lncRNA *MAFG‐AS1*, which is highly expressed in BUC, is correlated with aggressive characteristics and poor prognosis of BUC. We demonstrate that *MAFG‐AS1* can promote BUC proliferation, invasion, metastasis, and epithelial‐mesenchymal transition in vitro and in vivo. Mechanistically, *MAFG‐AS1* direct binding to Hu antigen R (HuR) could recruit ubiquitin‐specific proteinase 5 (USP5) to prevent HuR from degrading by ubiquitination. We further demonstrate that overexpression of *MAFG‐AS1* can upregulate the expression of polypyrimidine tract‐binding protein 1 (PTBP1) through promoting its stability mediated by bound HuR. In conclusion, these findings indicate that *MAFG‐AS1* promotes the progression of BUC via regulation of the HUR/PTBP1 axis. Targeting *MAFG‐AS1* may provide a novel strategy for individualized therapy and a potential biomarker for prognosis of BUC.

## Graphical abstract

Our findings indicate that MAFG‐AS1 acts as an oncogene to promote the progression of BUC via regulation of the HUR/PTBP1 axis. Targeting MAFG‐AS1 may provide a novel strategy for individualized therapy and a potential biomarker for prognosis of BUC

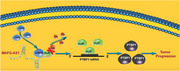

**Abbreviations**
ARE: AU‐rich elements
BUC: bladder urothelial carcinoma
ChIRP: chromatin isolation by RNA purification
CHX: cycloheximide
Co‐IP: co‐immunoprecipitation
FISH: fluorescence in situ hybridization
HuR: Hu antigen R
IHC: immunohistochemistry
IP: immunoprecipitation
lncRNA: long noncoding RNA
MIBC: muscle invasive bladder cancer
miRNA: micro‐RNA
NMIBC: nonmuscle invasive bladder cancer
PRM: parallel reaction monitoring
PTBP1: polypyrimidine tract‐binding protein 1
RBP: RNA‐binding protein
RIP: RNA immunoprecipitation
RT‐qPCR: quantitative real‐time polymerase chain reaction
TNM: tumor‐node metastasis
USP5: ubiquitin‐specific processing protease 5

## 1 INTRODUCTION

As the most prevalent malignant neoplasm that occurs in the urinary system, bladder urothelial carcinoma (BUC) has experienced an increasing incidence and mortality rate in recent years. There are an estimated 430 000 BUC new cases, and 165 000 deaths globally every year.^1^ BUC is divided into two classifications: nonmuscle invasive bladder cancer (NMIBC), which accounts for 70‐80% of cases, and muscle invasive bladder cancer (MIBC), which represents the remaining cases. Due to the progressiveness and distant metastasis of NMIBC, nearly 75% of high‐grade NMIBC end with relapse, progression, or death within 5 years. Despite significant improvements in radical cystectomy, neoadjuvant chemotherapy, and radiotherapy for MIBC, the mortality rate is up to 77.6% within 5 years of diagnosis.^2^, ^3^ Therefore, the progression, extensive metastasis, and recurrence of BUC remain major challenges, and the investigations of molecular mechanisms that contribute to these phenomena is urgently required.

Long noncoding RNAs (lncRNAs) are RNAs longer than 200 nucleotides with no or limited ability to encode proteins,^4^ and can promote tumor proliferation, invasion, and migration through various mechanisms to affect tumor development.^5^, ^6^, ^7^, ^8^ For example, lncRNAs may act as sponges by absorbing micro‐RNA (miRNA), or regulate neighboring genes in cis by recruiting regulatory factors to the locus and regulating their functions. Moreover, lncRNAs can also bind proteins to promote their functions by preventing their degradation and increasing their stability. In recent years, there has been increasing evidence showing that the interactions between lncRNAs and proteins play an important role in carcinogenesis and metastasis.^9^, ^10^, ^11^, ^12^, ^13^, ^14^ For example, BLACAT2 can bind with WDR5 to promote lymphatic metastasis through upregulating VEGF‐C expression in BUC.^15^ Additionally, Wang et al^16^ have confirmed that lncRNA EPIC1 can directly bind to Myc and form a positive feedback loop to stimulate tumorigenesis in breast cancer. However, further clarification of the molecular interactions between lncRNAs and proteins is required, and this may contribute to understanding the progressiveness and distant metastasis in BUC.

RNA‐binding proteins (RBPs) are proteins that interact with RNA molecules to regulate the production, maturation, and localization of RNA in cells as well as their translation and degradation. Some studies have reported that dysregulated RBPs can lead to tumorigenesis by affecting the expression of genes. Hu antigen R (HuR), also known as ELAVL1, a member of ELAV‐like protein 1 family, is a ubiquitously expressed RBP that binds to AU‐rich elements (ARE) in the 3′UTR of mRNA.^6^, ^17^ Reports have shown that HuR could function as an oncogene in tumors,^18^, ^19^, ^20^, ^21^ and that increased HuR expression contributes to carcinogenesis and poor prognosis in BUC.^22^, ^23^ However, the precise mechanisms of HuR involvement in tumorigenesis of BUC remain elusive.

Here, we determined that *MAFG‐AS1* is the only lncRNA that is highly expressed in BUC according to both the GSE31189 and TCGA databases. High expression of *MAFG‐AS1* is correlated with aggressive cancer characteristics and poor prognosis. Furthermore, we found that *MAFG‐AS1* can directly bind to HuR and stabilize it by recruiting the deubiquitinating enzyme, ubiquitin‐specific processing protease 5 (USP5). Moreover, we demonstrate that *MAFG‐AS1* promotes malignant phenotypes in BUC by upregulating polypyrimidine tract‐binding protein 1 (PTBP1) via direct binding to and stabilization of HuR. Taken together, we reveal that the *MAFG‐AS1*/HuR/PTBP1 axis facilitates carcinogenesis and progression in BUC, which could provide new targets and strategies for the therapy of BUC.

## 2 MATERIALS AND METHODS

### 2.1 Bioinformatics analysis

lncRNA expression profiles in human BUC tissues were derived from the Cancer RNA‐Seq Nexus,^24^ TANRIC,^25^ and Xena databases. GEPIA and UALCAN^26^ databases were used for survival analysis. GEO (GSE31189, GSE87304, GSE13507, GSE31684, GSE124305), ChIPBase 2.0, and starBase 2.0 databases were used to predict the correlation between RBPs and lncRNAs. catRAPID,^27^ starBase 2.0, HDOCK,^28^ and POSTAR2.0 databases were used to predict the possible binding sites of RBPs on lncRNA. Gene ontology (GO) analysis was conducted using the Metascape database. A list of RNA degradation genes was downloaded from the Molecular Signatures Databases^29^ (KEGG_RNA_DEGRADATION, M963). Gene set enrichment analysis (GSEA) was performed using the GSEA software. All databases used are listed in Table S7.

### 2.2 Tissues and cell lines

For *MAFG‐AS1* expression profiling analysis, a cohort of BUC tissues was derived from 102 patients with BUC who had undergone radical cystectomy at the Sun Yat‐sen University Cancer Center (Guangzhou, Guangdong, China) and Third Xiangya Hospital of Central South University (Changsha, Hunan, China) from 2007 to 2015. The tumor‐node metastasis (TNM), grade, and stage were classified with the World Health Organization and American Joint Committee on Cancer. The clinicopathologic characteristics of these patients are summarized in Table S1. An additional panel of six formalin‐fixed, paraffin‐embedded tissues, snap‐frozen fresh BUC tissues, and matched adjacent normal bladder tissues were obtained from patients who underwent radial cystectomy at the Third Xiangya Hospital of Central South University between 2016 and 2018. These clinical specimens were previously approved for research use by the patients and the ethics committee. The fresh tissue samples were quantified by quantitative real‐time polymerase chain reaction (RT‐qPCR) and/or Western blotting as described below. We purchased human BUC cells (5637, BIU87, EJ, RT4, and T24 cells) from ATCC (Rockville, MD), and cultured in DMEM (Invitrogen, CA), added with 10% fetal bovine serum (FBS) (GIBCO, NY), 1 mmol/L glutamine, and 1% penicillin/streptomycin.

**Highlights**

1. lncRNA *MAFG‐AS1* is correlated with aggressive characteristics of BUC;
2. BUC patients with high levels of *MAFG‐AS1* have poor prognosis;
3. *MAFG‐AS1* direct binding to HuR could recruit USP5 to prevent HuR from degrading by ubiquitination;
4. *MAFG‐AS1* promotes malignant phenotypes by upregulating of PTBP1 via direct binding to and stabilization of HuR in BUC.

### 2.3 Cell transfection

Plasmids for overexpression *MAFG‐AS1* were purchased from GeneChem Biotechnology (Shanghai, China). Three *MAFG‐AS1*‐specific si‐RNAs (si‐RNA1, si‐RNA2, and si‐RNA3), si‐HuR, si‐PTBP1, si‐USP5, si‐UCHL5, si‐COPS6, si‐PSMD14, si‐OTUB1, and shRNAs targeting *MAFG‐AS1*, HuR, PTBP1, and negative control were purchased from GeneChem Biotechnology. The T24 and RT4 cells were transfected the recombinant plasmid using Lipofectamine 3000 (Invitrogen). RT‐qPCR was performed to determine the transfection efficiency after 48‐72 hours of transfection.

### 2.4 RT‐qPCR

Total RNA was extracted from BUC tissues and cells using TRIzol (Invitrogen) and the purity of the RNA was assessed spectrophotometrically (A260/A280 > 1.8). Total RNA (500 ng) was subjected into cDNA using the MMLV reverse transcriptase enzyme (Promega, Madison, WI). RT‐qPCR was performed using SYBR Green PCR Master Mix (Life Technologies Corporation, CA). Gene expression was normalized to that *GAPDH* mRNA or U1 snRNA. The reactions were performed independently and in triplicate. The 2^−ΔΔCt^ method was applied to calculate relative expression levels of genes. All primers used are listed in Table S8.

### 2.5 Wound‐healing and transwell assays

The migration capacity of BUC cells was measured by wound‐healing assays as described previously.^30^ Briefly, BUC cells were cultivated in a six‐well plate. The cellular monolayers were wounded by scratching with a 20 μL pipette tip when cultures reached >90% confluency. Images were photographed at 0, 24, and 48 hours after wounding. The invasiveness of cells assessed using a transwell assay. BUC cells (5 × 10^4^) were plated into a transwell plate of 8 μm pores (Corning Inc, Corning, NY). The two layers of the transwell contained two different media compositions: DMEM medium containing 1% FBS (upper transwell chamber), and DMEM containing 15% FBS (lower transwell chamber). The invasive cells were fixed with 95% ethanol, stained with hematoxylin (after 24 hours incubation), and then counted using an inverted microscope.

### 2.6 MTT assay

Transfected BUC cells were inoculated in 96‐well plates and incubated for 24, 48, 72, and 96 hours at 37°C and 5% CO_2_, then MTT (50 μL) (Sigma Chemicals, MO) was added to each well and incubated for an additional 4 hours. Supernatants were removed from cells by aspiration and DMSO was added. Finally, the absorbance at 570 nm was measured.

### 2.7 Clone formation assay

BUC cells, 48 hours posttransfection were inoculated into a six‐well plate and cultured at 37°C, 5% CO_2_, and saturated humidity for 2‐3 weeks. Cell colonies were fixed with 4% paraformaldehyde and stained with hematoxylin. Fixed and stained cells were counted to quantify clone formation. If the number of cloned cells added up to 50, it was counted as a clone.

### 2.8 Western blotting

Western blotting was performed as described previously.^31^, ^32^ Briefly, tissues and cells were lysed on ice for 30 minutes using RIPA lysis buffer (Beyotime, Shanghai, China) containing 10% protease inhibitor cocktail (Roche, IN), and the protein concentration was quantified with a BCA Protein Assay Kit (Thermo Scientific). Subsequently, the protein samples were separated on a 10% SDS‐polyacrylamide gel and transferred to PVDF membrane. The membranes were blocked in 5% skimmed milk powder in PBS and incubated overnight anti‐HuR (Proteintech, 11910‐1‐AP), anti‐PTBP1 (Abcam, ab5642), anti‐UCHL5 (Abcam, ab124931), anti‐USP5 (Proteintech, 10473‐1‐AP), anti‐COPS6 (Abcam, ab77299), anti‐PMSD14 (Proteintech, 12059‐1‐AP), anti‐OTUB1 (Abcam, ab233160), and anti‐GAPDH (Abcam, ab125247) at 4°C with. This was followed by 30‐60 minutes incubation with secondary antibody. ECL was used to visualize the signals of protein bands and quantified using the optical density analysis software Quantity One (Bio‐Rad). GAPDH protein levels were used as internal control.

### 2.9 Xenograft mouse model

Five‐week‐old female BALB/c nude mice weighing 18‐22 g was purchased from Shanghai Laboratory Animal Center (SLAC, Shanghai, China). Briefly, 1 × 10^6^ of overexpressing *MAFG‐AS1* T24/RT4 cells, sh‐*MAFG‐AS1* T24/RT4 cells, *MAFG‐AS1 *+ sh‐HuR T24/RT4 cells, and *MAFG‐AS1 *+ sh‐PTBP1 T24/RT4 cells were injected subcutaneously into mice. Tumor volumes were calculated using the equation (length × width^2^)/2 every 3 days. Nude mice were euthanized at day 25. For lung metastasis models, 2 × 10^6^ of T24/RT4 cells were injected into mice via the tail vein. The tumors and lungs were stained with hematoxylin and eosin (H&E) at day 45. The number of lung metastatic nodules in each mouse was calculated. All experimental procedures were approved by the University Ethics Committee.

### 2.10 Immunohistochemistry (IHC)

IHC staining was performed following similar methods to those previously published.^31^ Cancer tissue sections were deparaffinized, then blocked with 1% PBA. The prepared sections were incubated with anti‐HuR (Proteintech, 11910‐1‐AP), anti‐PTBP1 (Abcam, ab5642), and anti‐Ki67 (Genetex, GTX 16667) at 4°C overnight. The incubated polymer enhancers were then added with a biotin‐labeled secondary antibody. Next, the sections were stained with a DAB staining solution, and counterstained with hematoxylin. Two independent pathologists assessed the IHC scores in a blinded manner; the details of the scoring methodology have been previously published.^31^

### 2.11 Fluorescence in situ hybridization (FISH)

The Cy3‐labeled *MAFG‐AS1* probe and U6 RNA were designed and synthesized by RiboBio (Guangzhou, China). FISH kit (RiboBio) was used to implement the FISH experiments as per the manufacturer's instructions.

### 2.12 Immunoprecipitation (IP)

#### 2.12.1 RNA immunoprecipitation (RIP)

The RIP assay was performed using the EZ‐Magna RIP kit (Millipore, MA). Briefly, collected and incubated BUC cells (1 × 10^7^) with RIP lysis buffer. The precleared lysates were used for RIP with anti‐HuR (Proteintech, 11910‐1‐AP) and rabbit isotype control IgG antibodies. RNA was isolated and purified using an acid phenol/chloroform method.

#### 2.12.2 Co‐immunoprecipitation (Co‐IP)

Th**e** growth medium of adherent cells in a 10 cm cell culture dish was removed by aspiration and washed once with PBS. The cells were then lysed with 1 mL IP buffer, mixed with 2 μg anti‐HuR (Proteintech, 11910‐1‐AP) and incubated with rotation overnight at 4°C. Protein A/G Agarose was fully resuspended in 20 μL buffer A and shaken slowly. IP complex was washed in IP buffer five times and 20 μL of 1 × SDS‐PAGE loading buffer was added and mixed. The pellet was vortexed and then centrifuged to concentrate the sample at the bottom of the tube. Western blot analysis was performed using antiubiquitin (Proteintech, 10201‐2‐AP) and anti‐USP5 (Proteintech, 10473‐1‐AP).

### 2.13 Chromatin isolation by RNA purification

In total, 1 × 10^9^ to 5 × 10^9^ cells were used for the chromatin isolation by RNA purification‐mass spectrometry (ChIRP‐MS) experiment. Briefly, after harvesting, cross‐linking, and lysis of cells, the DNA was sheared into small fragments through sonication. Lysed cells were transfected with a biotin‐labeled *MAFG‐AS1* probe or NC probe (Table S4). ChIRP was performed with the following modifications^33^: (a) cross‐linking cells in 3% formaldehyde for 30 minutes, and then quenched with 0.125 M glycine for 5 minutes; (b) for hybridization, 100 pmol probe was mixed with chromatin (1 mL) in Falcon tube (15 mL) and incubated at 37°C for 4 hours with shaking, stripped the beads from the buffer with a DynaMag‐2 magnet; (c) for MS, the beads were removed twice from precleared lysates using a magnetic stand; (d) for RNase control, the lysates were first pooled and mixed into two equal parts. Before hybridization, samples were incubated at 37°C for 30 minutes with mixing. Then, RNA extraction and protein elution were carried out as previously described.^34^ Finally, protein samples were size‐separated by a nano‐UPLC liquid phase system (EASY‐nLC1200) and used for MS.

### 2.14 Parallel reaction monitoring (PRM)

PRM‐MS analysis was as previously described.^35^ Tryptic polypeptides (5 μL) from each sample were dissolved in solution A (0.1% formic acid‐acetonitrile). Peptides were separated on a reversed‐phase chromatographic column (Reprosil‐Pur 120 C18‐AQ, 1.9 μm, Dr. Math) using the following gradient system at a flow rate of 300 nL/min: 92 minutes from 8% to 35% buffer B (98% acetonitrile and 0.1% formic acid), 20 minutes from 35% to 45% buffer B, 2 minutes from 45% to 100% buffer B, 2 minutes at 100% buffer B, and 2 minutes at 2% buffer B. PRM data were collected by MS and imported into Skyline (version 3.6.1) for transition extraction.

### 2.15 Stability and α‐amanitin treatment

We inoculated *MAFG‐AS1* shRNA, NC shRNA, with T24/RT4 cells overexpressing *MAFG‐AS1* and empty plasmid. Then treated with 50 μg/Ml α‐amanita toxin,^36^ and harvested after 24 hours. The cells were processed for RNA purification and RT‐qPCR after 6, 12, 18, 24, and 48 hours of treatment. Three independent samples were collected for each data point.

### 2.16 Statistical analysis

GraphPad Prism 8.0.2 was used for all statistical analyses, and all experiments were repeated three times. Data are expressed as mean ± standard deviation (SD). Significant differences between the two groups were analyzed using Student's *t*‐tests, whereas significant differences between more than two groups were analyzed using one‐way ANOVA followed by Dunnett's test. Survival curves were plotted using the Kaplan‐Meier method and compared with log‐rank tests. *P*‐value < .05 was considered to be a significant difference.

## 3 RESULTS

### 3.1 *MAFG‐AS1* is highly expressed and negatively correlated with prognosis in BUC

**FIGURE 1:**
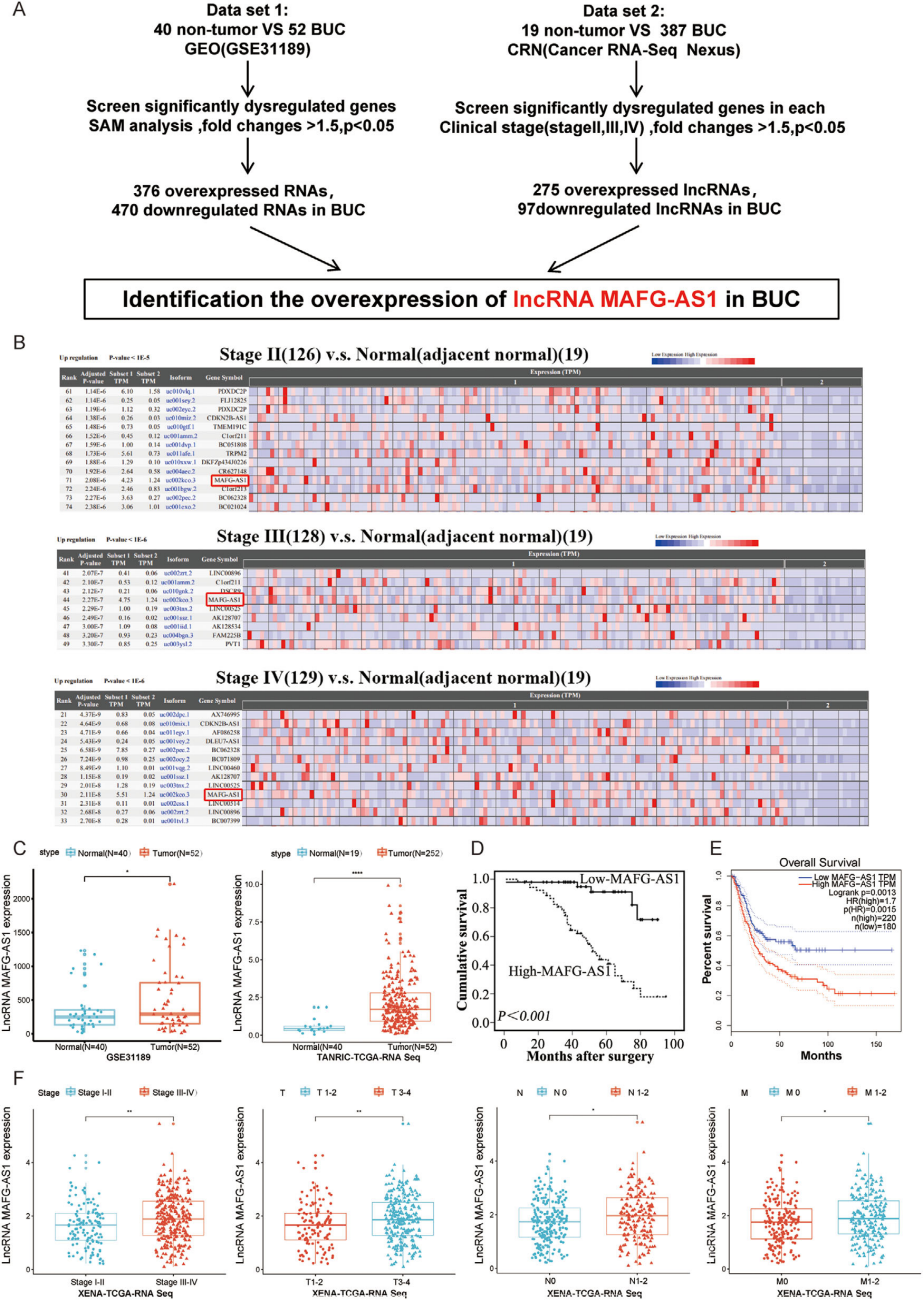
*MAFG‐AS1* is highly expressed and negatively correlated with prognosis in bladder urothelial carcinoma (BUC). A, Screening significantly dysregulated lncRNAs in BUC. B, Expression level of *MAFG‐AS1* in BUC patients with each stage; data from Cancer RNA‐Seq Nexus. C, The expression of *MAFG‐AS1* in cancer and normal tissues of BUC; data from GES31189 dataset and TANRIC database. D and E, The relationship between *MAFG‐AS1* and the prognosis of BUC. F, The expression of *MAFG‐AS1* in different stages of BUC patients. Bars represent standard deviation, ns *P* > .05, *P < .05, **P < .01, ***P < .001

The level of *MAFG‐AS1* was higher in BUC tissues than that in adjacent normal tissues using RT‐qPCR (Figure S1C). Moreover, analysis of both GEO (GSE31189) and TCGA databases revealed that *MAFG‐AS1* is the only lncRNA that is significantly upregulated in both databases (Figure 1A). *MAFG‐AS1* was significantly upregulated in BUC patients in clinical stages II, III, and IV compared with adjacent normal tissues (Figures 1B and 1C). Interestingly, *MAFG‐AS1* was upregulated in multiple cancers, for instance, breast cancer and colorectal adenocarcinoma (Figure S1A). We analyzed the expression of *MAFG‐AS1* to determine its potential clinical and prognostic significance in a cohort of 102 BUC tissues. We found that the higher levels of *MAFG‐AS1* were correlated with advanced T and N stages (Table S1). Moreover, BUC patients with highly expressed MAFG‐AS1 were associated with poorer prognosis by Kaplan‐Meier survival analysis (Figure 1D). In univariate analyses, M*AFG‐AS1* levels, T status, and N status were notably associated with survival of BUC patients (Table S2). Further multivariate analysis revealed that *MAFG‐AS1* and N status were independent prognostic indicators for survival (Table S3). Additionally, the expression level of *MAFG‐AS1* in BUC patients with stage III‐IV BUC was higher than that in stage II patients (Figure S1B). The relative expression of *MAFG‐AS1* in BUC patients at different stages was further verified by analyzing the TANRIC database. As shown in Figure 1F, the expression level of *MAFG‐AS1* was markedly higher in advanced clinical stages (III‐IV, N = 292), advanced T stages (T3‐4, N = 269), advanced N stages (N1‐2, N = 174), and advanced M stages (M1‐2, N = 219) of BUC patient tissues, compared to those with earlier clinical stages (I‐II, n = 132), T stages (T1‐2, n = 124), N stage (N0, n = 246), and M stage (M0, N = 204), respectively. Furthermore, the GEPIA dataset indicated that patients with high *MAFG‐AS1* expression levels not only had a shorter overall survival but also a shorter disease‐free survival (Figure 1E; Figure S1D). Altogether, these data suggested that *MAFG‐AS1* was highly expressed in BUC, which associated with aggressive characteristics and poor prognosis, further supporting the oncogenic role of *MAFG‐AS1* in BUC.

### 3.2 *MAFG‐AS1* can promote proliferation, invasion, metastasis, and EMT of BUC cells

**FIGURE 2:**
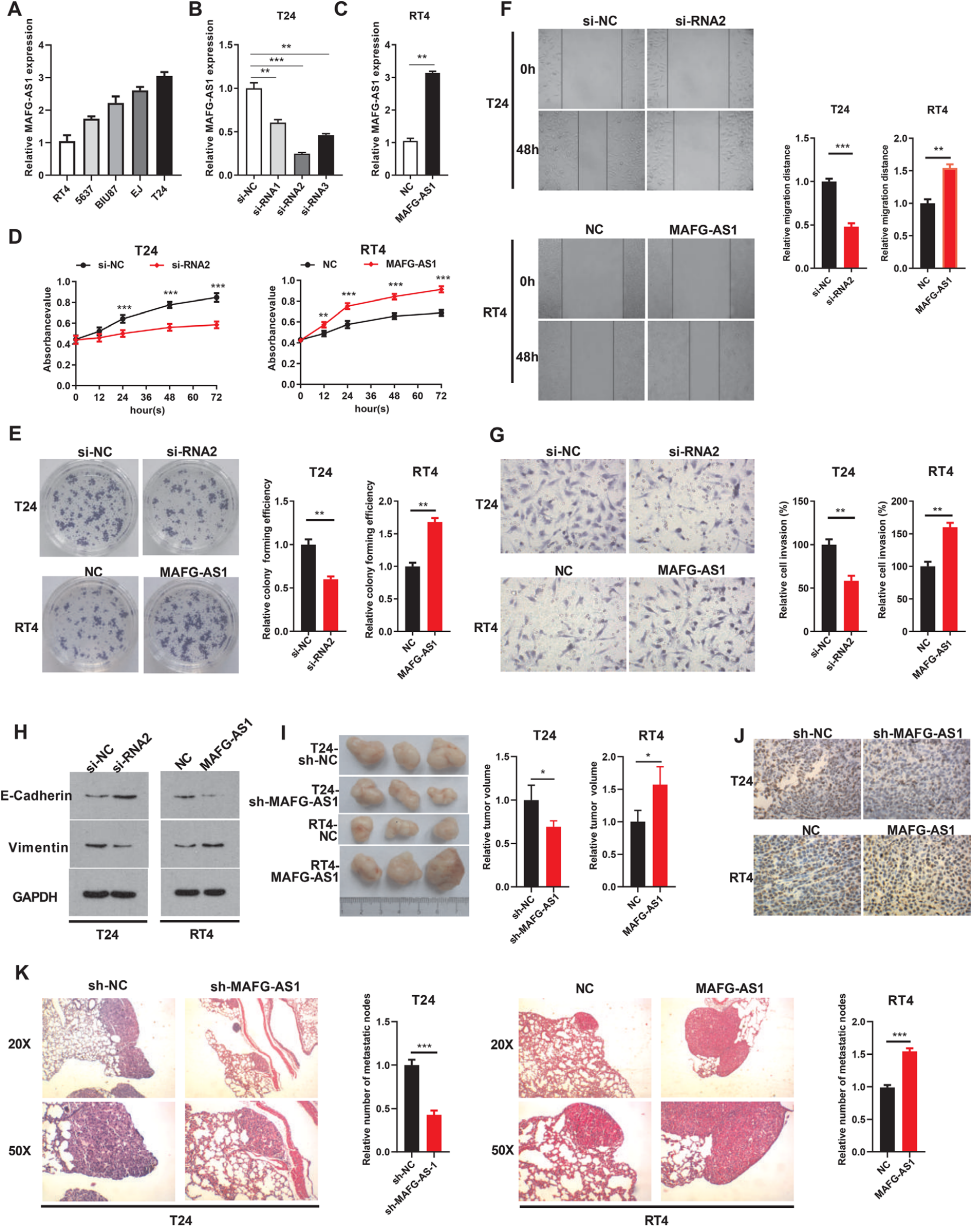
*MAFG‐AS1* can promote the proliferation, invasion, metastasis, and epithelial‐mesenchymal transition (EMT) of bladder urothelial carcinoma (BUC) cells. A, The expression level of *MAFG‐AS1* in different BUC cell lines (BIU87, 5637, T24, EJ, and RT4) was detected by RT‐qPCR. B and C, The expression level of *MAFG‐AS1* in T24/RT4 cells was detected by RT‐qPCR after transfection of si‐RNA and overexpression plasmid. D and E, MTT (D) and clone formation assays (E) showed the proliferation of T24 and RT4 cells with knockdown or overexpression of *MAFG‐AS1*. F and G, Wound‐healing (F) and transwell assays (G) were performed to evaluate cell migration and invasion in T24 and RT4 cells treated with knockdown or overexpression of *MAFG‐AS1*. H, Western blot analysis showed the expression level of EMT‐related proteins. I, Images of tumor xenografts from nude mice. J, Immunohistochemistry to detect the expression level of Ki67 in tissues. K, Representative images of lung metastatic foci after hematoxylin and eosin (H&E) staining. Bars represent standard deviation, ns *P* > .05, *P < .05, **P < .01, ***P < .001

Since the upregulated *MAFG‐AS1* is closely related to the prognosis of patients, we further explored the function of *MAFG‐AS1* in BUC. We examined the expression of *MAFG‐AS1* in five BUC cell lines and found that the expression of *MAFG‐AS1* was relatively higher in T24 cells compared with RT4 cells through RT‐qPCR (Figure 2A). Subsequently, RT4 and T24 BUC cell lines were established to stably overexpress *MAFG‐AS1* and siRNA targeting *MAFG‐AS1* (Figures 2B and 2C). The proliferative capacities of cells were strikingly suppressed after knockdown of *MAFG‐AS1* by clone formation and MTT assays (Figures 2D and 2E). The influence of *MAFG‐AS1* on invasion and metastasis was investigated by transwell and wound‐healing assay. Similarly, the invasion and metastatic ability of BUC cells was weakened after knocking down *MAFG‐AS1* (Figures 2F and 2G). On the contrary, overexpression of *MAFG‐AS1* dramatically elevated cell proliferation, migration, and invasion in BUC cells (Figure 2D‐G). Meanwhile, E‐cadherin, the epithelial marker, was increased after silencing *MAFG‐AS1*, while the expression of vimentin, the mesenchymal marker, was increased in *MAFG‐AS1‐*overexpressing cells, indicating that *MAFG‐AS1* contributed to epithelial‐mesenchymal transition (EMT) progression in BUC (Figure 2H). Additional studies were conducted to verify the effects of *MAFG‐AS1* on subcutaneous tumor formation and distant lung metastasis in BUC cells. Tumor volume was significantly reduced in the sh‐*MAFG‐AS1* group compared with the control group (Figure 2I). The positive rate of proliferation index Ki67 (Figure 2J) and the number of lung metastatic nodules (Figure 2K) were significantly reduced after silencing MAFG‐AS1, while overexpression of *MAFG‐AS1* exerted promoting effects on the tumor volume (Figure 2I), proliferative ability (Figure 2J), and lung metastatic capacities (Figure 2K). These results indicated that upregulation of *MAFG‐AS1* considerably promoted progression of BUC both in vivo and in vitro.

### 3.3 *MAFG‐AS1* binds directly to HuR

**FIGURE 3:**
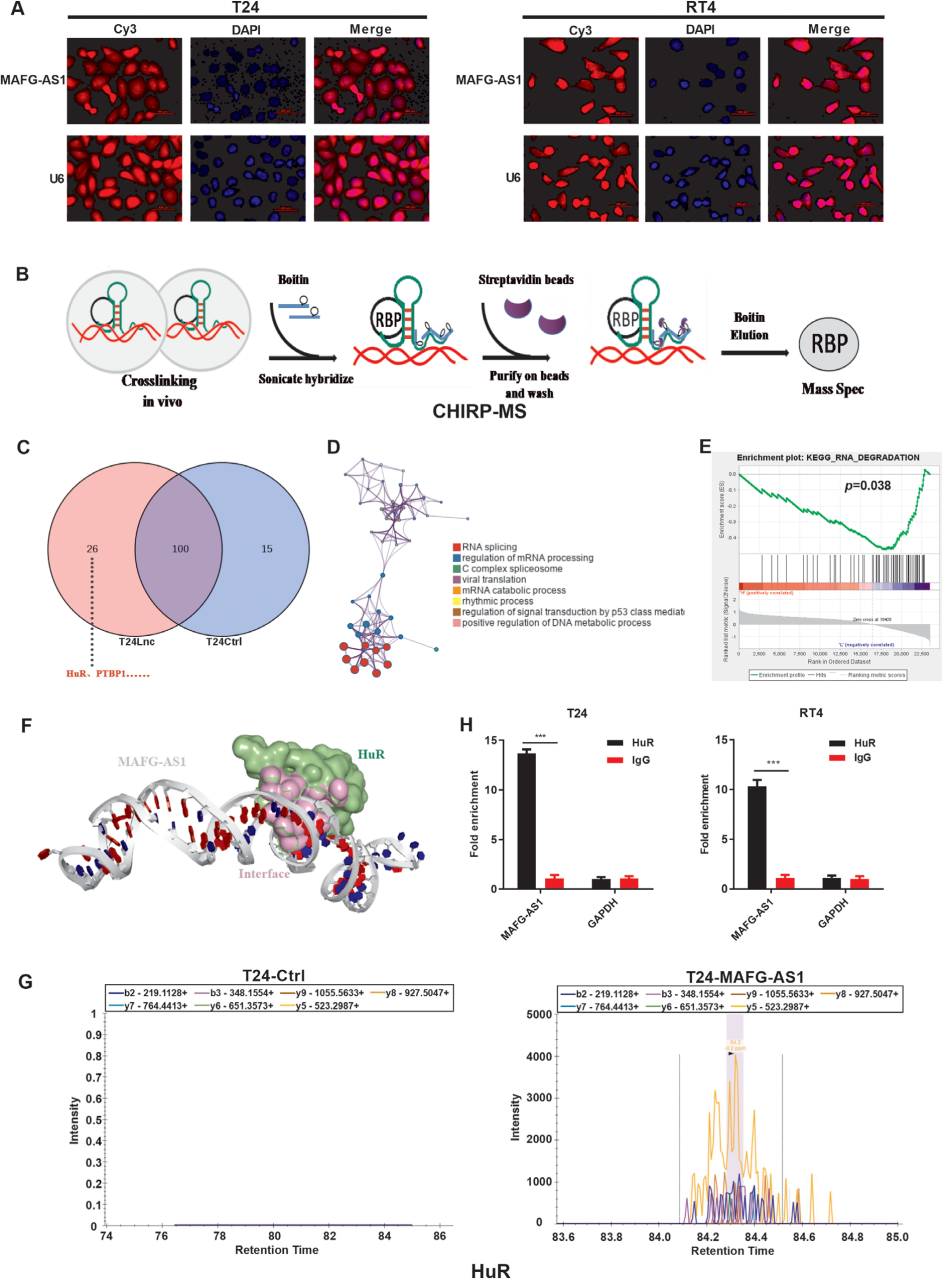
*MAFG‐AS1* binds directly to Hu antigen R (HuR). A, FISH for detecting the localization of *MAFG‐AS1* in T24 and RT4 cells. B, Schematic diagram of the ChIRP experiment. C, ChIRP‐MS detects the binding proteins of *MAFG‐AS1*. D, Gene ontology (GO) analysis results. E, The GSEA analysis results (187 gene sets were used for the GSEA). F, HDOCK database predicts that *MAFG‐AS1* binds to HuR. G, PRM assay validates proteins that *MAFG‐AS1* may bind to. H, RIP assay to verify that HuR binds to *MAFG‐AS1*. Bars represent standard deviation, ns *P* > .05, *P < .05, **P < .01, ***P < .001. DAPI, 4ʹ,6‐diamidino‐2‐phenylindole; T24‐Lnc, T24‐*MAFG‐AS1*; T24‐Ctrl, T24‐negative control

To identify the location of *MAFG‐AS1*, RNA FISH was performed. The results revealed that *MAFG‐AS1* was enriched in not only cytoplasm but also in nucleus of T24 and RT4 cells (Figure 3A). Taking into account that lncRNAs can bind with proteins to promote tumor development, we then screened for the binding protein of *MAFG‐AS1* using ChIRP‐MS assay (Figure 3B). The results showed that the specific probe group of *MAFG‐AS1* bound to 26 proteins (Figure 3C; Table S5). We then performed GO analysis based on only these 26 genes. The GO analysis showed that these genes were related to the regulation of mRNA processing (Figure 3D). GSEA suggested that MAFG‐AS1 is involved in RNA degradation (Figure 3E).

HuR, a member of ELAVL Family, is an RBP involved in a variety of biological processes including RNA degradation. The HDOCK and catRAPID web servers predicted that HuR has binding sites for *MAFG‐AS1* (Figure 3F; Figure S4A). Furthermore, ChIRP‐MS data were further verified using PRM, which detected a strong signal of HuR in the protein pulled down by the *MAFG‐AS1*‐specific probe (Figure 3G; Table S6). To confirm the binding between *MAFG‐AS1* and HuR, RIP was performed and showed that *MAFG‐AS1* was evidently enriched in HuR group compared to the IgG control group (Figure 3H), indicating that *MAFG‐AS1* could bind to HuR directly. However, the consequences induced by this binding and whether the oncogenic role of *MAFG‐AS1* is associated with this interaction remain unknown.

### 3.4 *MAFG‐AS1* can stabilize HuR by recruiting the deubiquitinating enzyme USP5

**FIGURE 4:**
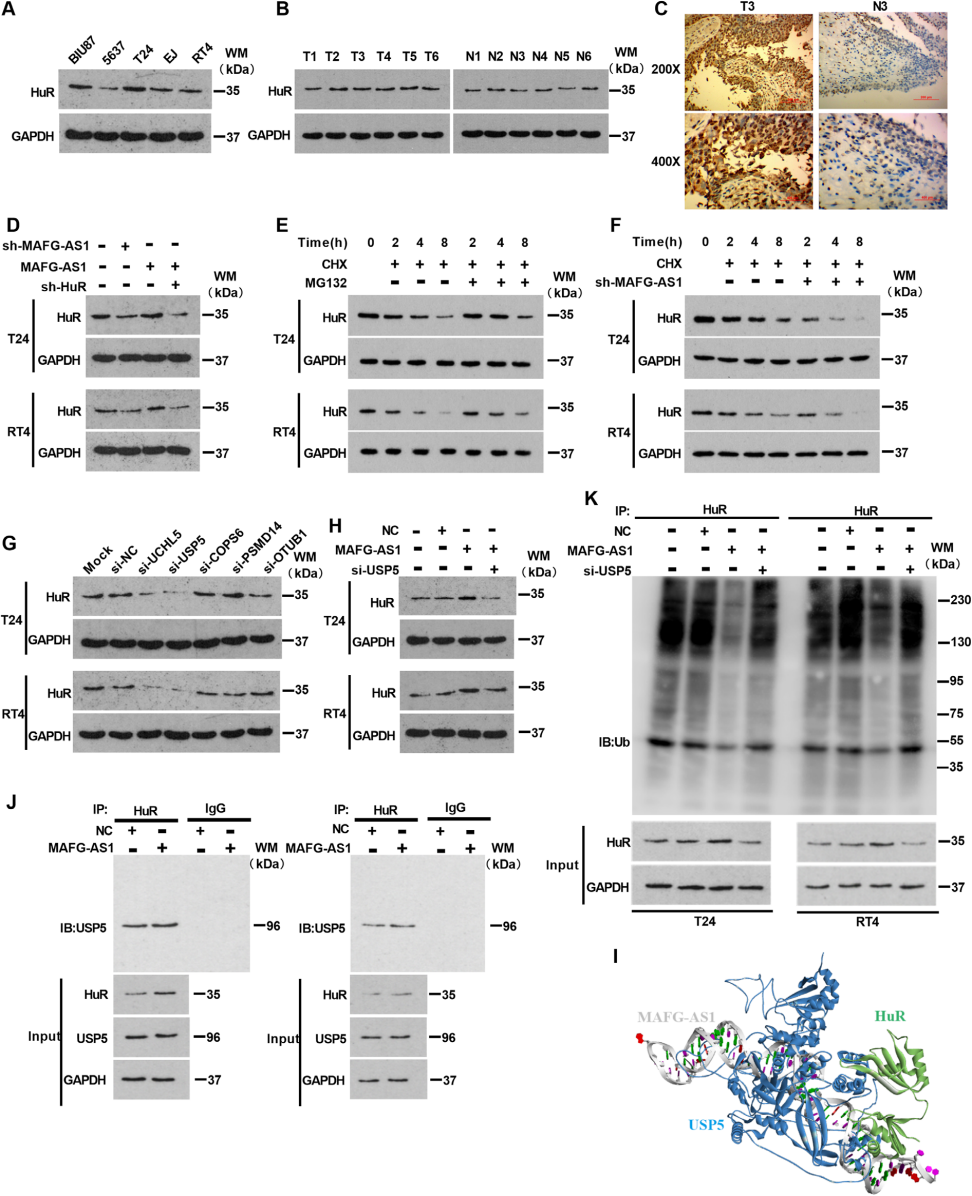
*MAFG‐AS1* can stabilize Hu antigen R (HuR) by recruiting the deubiquitinating enzyme USP5. A, Western blot analysis of HuR expression in bladder urothelial carcinoma (BUC) cells. B, Western blot analysis of HuR expression in cancer and normal tissues of bladder. C, Immunohistochemical detection of HuR expression in cancer and normal tissues of bladder. D, Western blotting was used to detect the expression level of HuR in each experimental group. E and F, T24 and RT4 cell lines were cultured with cycloheximide (10 μg/mL), adding MG132 (20 μM) or transfected with sh‐*MAFG‐AS1*, and cell lysates were analyzed by Western blotting. G, Detection of HuR protein expression by Western blotting after knockdown of deubiquitinating enzyme in T24 and RT4 cell lines. H, Western blotting shows the expression level of HuR in T24 and RT4 cells after transfected with *MAFG‐AS1* or *MAFG‐AS1* and si‐USP5. I, The HDOCK website predicts that HuR can bind to USP5. J, USP5 expression was determined by Co‐IP using HuR‐antibody to precipitate binding proteins. K, The level of ubiquitination of HuR was detected by Co‐IP after overexpression of *MAFG‐AS1* or overexpression of *MAFG‐AS1* and knocking down USP5 in T24/RT4 cells. Bars represent standard deviation, ns *P* > .05, *P < .05, **P < .01, ***P < .001

To identify the specific relationship between *MAFG‐AS1* and HuR, we conducted Western blotting in BUC cell lines (Figure 4A). Moreover, the expression of HuR was substantially upregulated in cancer tissues compared to noncancerous tissues (Figure 4B), which was further verified by IHC (Figure 4C). It was found that *MAFG‐AS1* was positively correlated with HuR using the ChIPBase 2.0 database (Figure S3A). Knockdown of *MAFG‐AS1* resulted in a notable reduction of HuR; however, HuR expression level was significantly increased when *MAFG‐AS1* was overexpressed in T24 and RT4 cells (Figure 4D). Therefore, we hypothesized that *MAFG‐AS1* might affect the stability of HuR. To verify this hypothesis, we treated T24 and RT4 cells with cycloheximide (CHX), an intracellular protein synthesis inhibitor, with or without proteasome inhibitor MG132. The results showed that HuR protein degradation was effectively slowed down in the MG132‐treated group, suggesting that HuR protein was affected by the ubiquitinated protease system (Figure 4E). Then, *MAFG‐AS1* was knocked down in T24 and RT4 cells with CHX. Similarly, knockdown of *MAFG‐AS1* reduced the stability of HuR compared with the control group (Figure 4F).

It is generally recognized that ubiquitination is a posttranslational modification that can regulate proteins’ functions, such as protein stability, subcellular localization, and activity. lncRNAs have been reported to participate in protein degradation through the ubiquitin proteasome system. Interestingly, it was predicted that there were multiple ubiquitinated sites in HuR using PhosphoSitePlus website (https://www.phosphosite.org//). Therefore, we inferred that *MAFG‐AS1* increased the stability of HuR via inhibition of ubiquitination. Next, we selected five deubiquitinating enzymes (UCHL5, USP5, COPS6, PSMD14, and OTUB1) that were positively correlated with *MAFG‐AS1* or associated with poor prognosis in BUC using the UALCAN database (Figure S2A‐J). Among the selected enzymes, knockdown of USP5 repressed the protein level of HuR the most (Figure 4G). Additionally, *MAFG‐AS1* was positively correlated with USP5 according to the GEO (GSE31684, GSE124305) and ChIPBase 2.0 database (Figure S3C,H,I). Western blotting analysis exhibited that HuR expression could be promoted by overexpression of *MAFG‐AS1*, but the effects could be weakened by cotransfection with *MAFG‐AS1* and si‐*USP5* (Figure 4H). Surprisingly, we found that HuR could bind to USP5 directly through analyzing both HDOCK and catRAPID (Figure S4D). Furthermore, *MAFG‐AS1* tended to bind with USP5 (Figure 4I; Figure S4B,C). We performed Co‐IP assays using anti‐HuR as a precipitating antibody to identify the relationship between USP5 and HuR in T24 and RT4 cells. As shown in Figure 4J, HuR directly binds to USP5. Moreover, the ubiquitination level of HuR was decreased in the *MAFG‐AS1‐*overexpressing cells, which could be reversed by cotransfection with *MAFG‐AS1* and si‐*USP5* in T24 and RT4 cells (Figure 4K). Taken together, *MAFG‐AS1* can stabilize HuR by recruitment of USP5, a deubiquitinating enzyme, thereby protecting HuR from degradation through ubiquitination.

### 3.5 *MAFG‐AS1* could promote PTBP1 translation by increasing its expression through enhancing HuR stability

**FIGURE 5:**
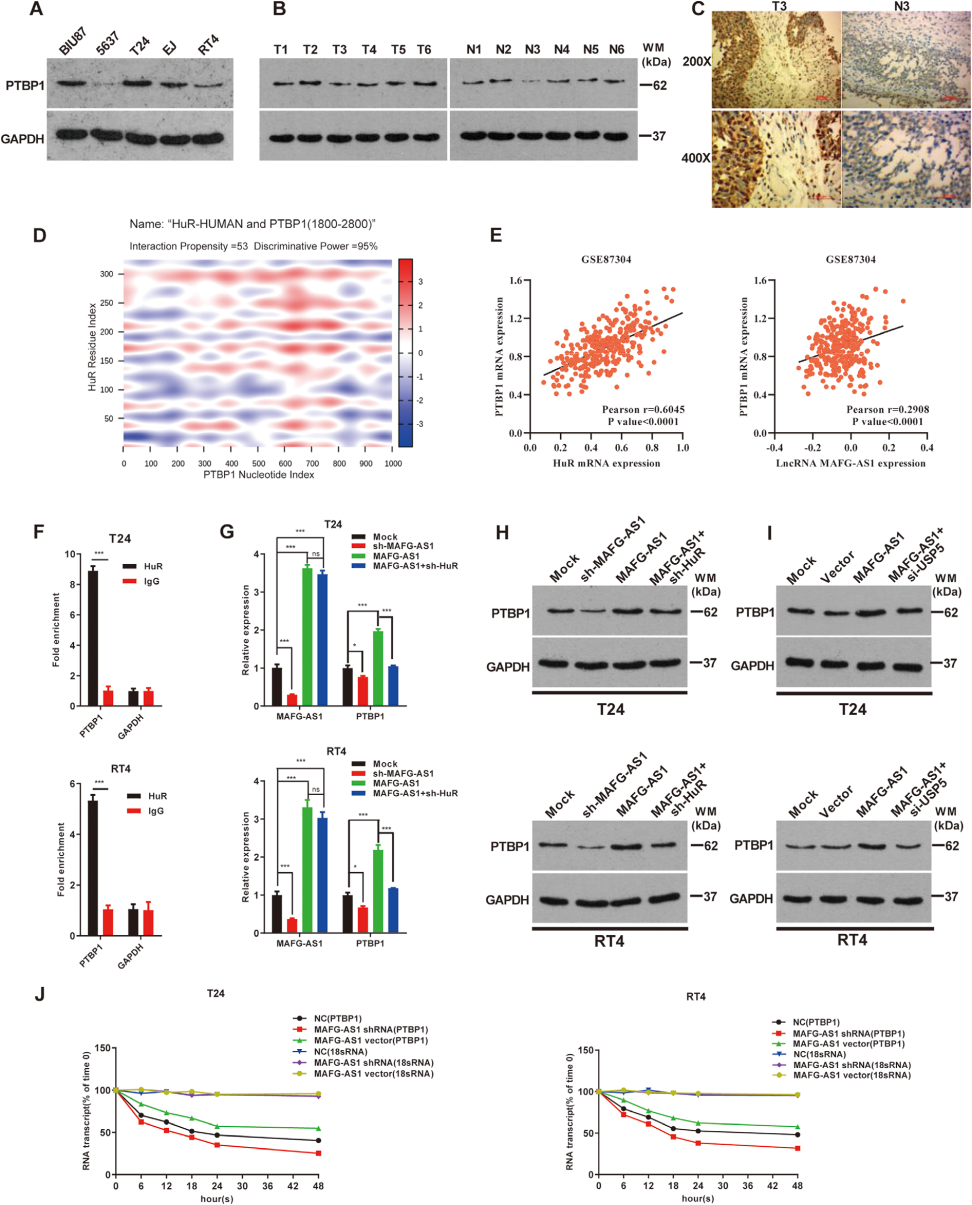
*MAFG‐AS1* could promote PTBP1 translation by increasing its expression through enhancing Hu antigen R (HuR) stability. A, The expression of PTBP1 protein was detected by Western blot in bladder urothelial carcinoma (BUC) cell lines (BIU87, 5637, T24, EJ, and RT4). B, Western blot assay detected the expression of HuR in tumor and adjacent tissues. C, Immunohistochemical detection of PTBP1 protein expression in tumor tissues and adjacent tissues of BUC patients. D, The catRAPID database predicted that HuR is much more likely to bind to *PTBP1* mRNA (discriminative power [DP] ranges from 0% (unpredictability) to 100% (predictability); DP values above 50% indicate that the interaction is likely to take place, whereas DPs above 75% represent high‐confidence predictions). E, HuR is positively correlated with PTBP1 in BUC based on the GEO dataset (GSE124305, GSE87304). F, RIP assay was conducted to detect whether HuR protein can bind to *PTBP1* mRNA. G, The effect of *MAFG‐AS1* and HuR on *PTBP1* mRNA levels was detected by RT‐qPCR. H and I, Western blot detected the expression of PTBP1 protein. J, The stability of *PTBP1* mRNA was measured by RNA stability assay in each experimental group. Bars represent standard deviation, ns *P* > .05, *P < .05, **P < .01, ***P < .001

To illustrate what effects could be induced by the binding between *MAFG‐AS1* and HuR, we analyzed several databases (such as catRAPID, starBase 2.0, and POSTAR2.0) to find out downstream effects of HuR. It was predicted that HuR, as an RBP, is highly likely to bind to PTBP1 (Figure 5D). PTBP1 was highly expressed in T24 cells with high metastatic invasion (Figure 5A), while RT4 cells with low metastasis levels exhibited decreased PTBP1 expression. Importantly, PTBP1 was upregulated in BUC tissues (Figure 5B). These results were further verified by IHC experiments (Figure 5C). Furthermore, HuR was coexpressed with PTBP1 at the transcriptional level based on data (GSE87304, GSE13507, GSE31684, GSE124305) from GEO and ChIPBase 2.0 databases (Figure 5E; Figure S3B,D‐F). The RIP assay indicated that the *PTBP1* mRNA could interact with HuR (Figure 5F). Therefore, we assumed that *MAFG‐AS1* could promote the expression of PTBP1 by enhancing HuR stability. The expression of *PTBP1* mRNA was increased significantly when *MAFG‐AS1* was overexpressed in T24 and RT4 cells (Figure 5G), and knockdown of HuR significantly inhibited *MAFG‐AS1* promotion of PTBP1 expression. Meanwhile, the expression of PTBP1 protein increased when *MAFG‐AS1* was overexpressed in T24 and RT4 cells, (Figure 5H), which could be reduced by knocking down USP5 or HuR (Figure 5I).

We intended to verify whether *MAFG‐AS1* regulates *PTBP1* mRNA stability. Thus, transfected of *MAFG‐AS1*‐vector or sh‐*MAFG‐AS1* into T24 and RT4 cells, with treatment of α‐amanitin, to inhibit transcription; 18S ribosomal RNA was used as an internal reference. Increased *PTBP1* mRNA level was observed after overexpression of *MAFG‐AS1*. Conversely, *MAFG‐AS1* knockdown shortened the half‐life of *PTBP1* mRNA (Figure 5J).

Overall, the results concluded that *MAFG‐AS1* could promote the expression of PTBP1 by promoting HuR stability.

### 3.6 *MAFG‐AS1*/HuR/PTBP1 axis can promote proliferation, invasion, and EMT of BUC in vivo and in vitro

**FIGURE 6:**
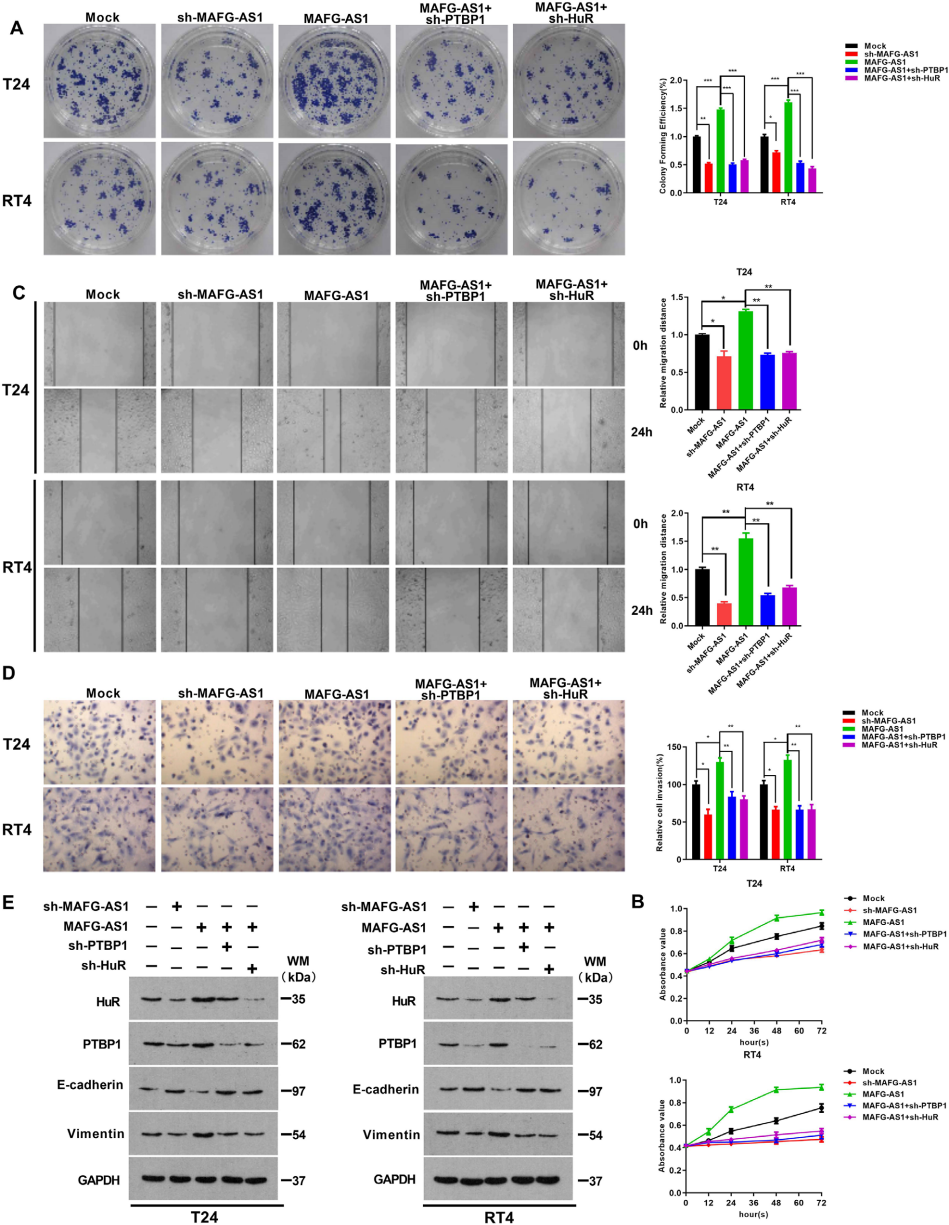
*MAFG‐AS1/*HuR/PTBP1 axis can promote proliferation, invasion, metastasis, and epithelial‐mesenchymal transition (EMT) of bladder urothelial carcinoma (BUC) in vivo. A and B, Colony formation assay (A) and MTT assay (B) were used to examine the effect of *MAFG‐AS1*/HuR/PTBP1 axis on cell proliferation. C and D, The effect of *MAFG‐AS1*/HuR/PTBP1 axis on cell metastasis and invasion was examined by wound‐healing assay (C) and transwell assay (D). E, The expression of HuR, PTBP1, E‐cadherin, and vimentin was examined by Western blot. Bars represent standard deviation, ns *P* > .05, *P < .05, **P < .01, ***P < .001

**FIGURE 7:**
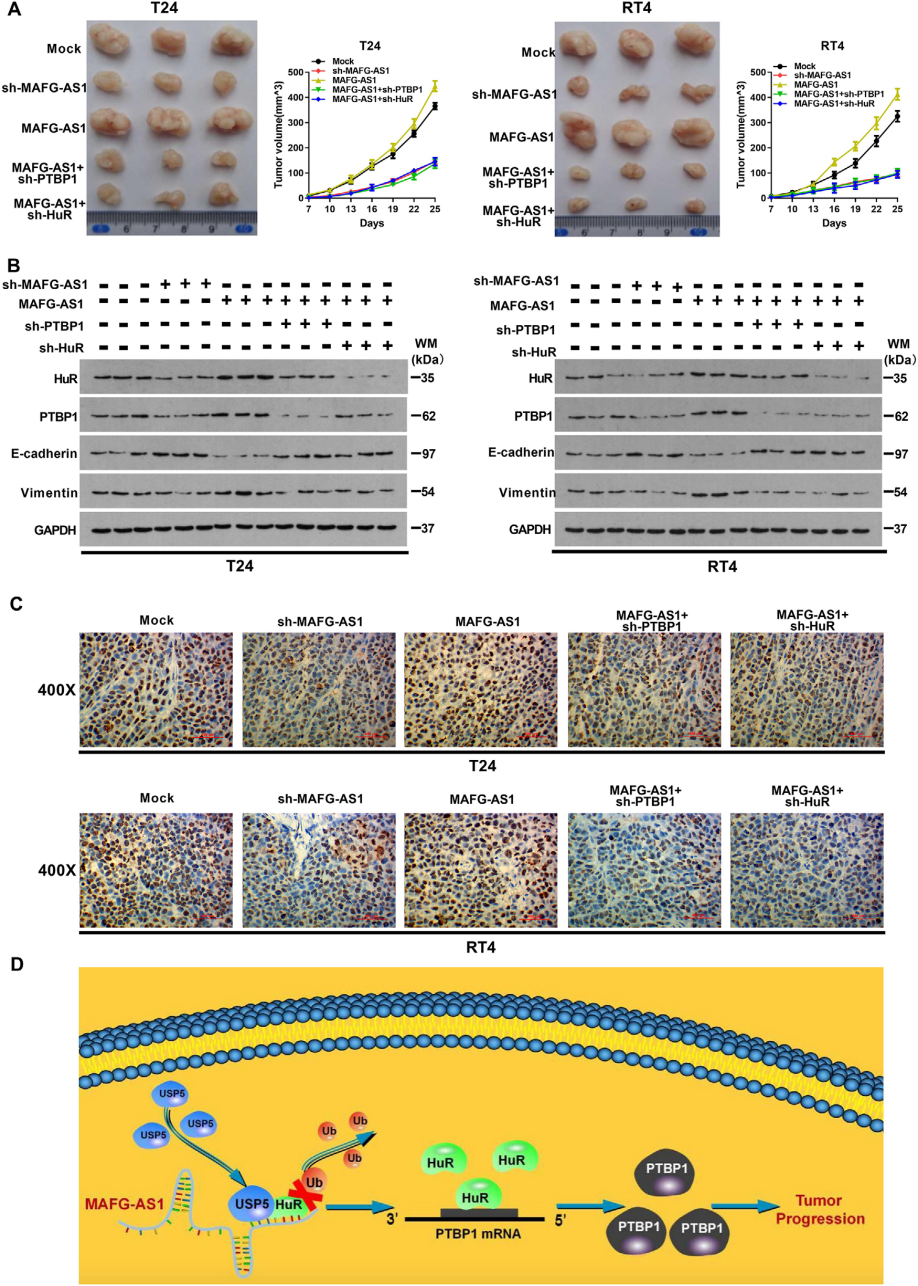
*MAFG‐AS1*/HuR/PTBP1 axis can promote proliferation, invasion, and epithelial‐mesenchymal transition (EMT) of bladder urothelial carcinoma (BUC) in vitro. A, Image of mouse xenograft in vivo assay and volume of tumor in each group. B, Western blot was used to detect the expression level of HuR, PTBP1, and EMT‐related proteins in tumor tissues of mice. C, Immunohistochemistry was used to detect the expression level of Ki67 in each group. D, Schematic diagram of *MAFG‐AS1*/HuR/PTBP1 axis promotes tumor progression in BUC. Bars represent standard deviation, ns *P* > .05, *P < .05, **P < .01, ***P < .001

We have identified that *MAFG‐AS1* promoted PTBP1 translation by increasing its stability through enhancing the expression of HuR in BUC. Next, to examine the biological role of that the *MAFG‐AS1*/HuR/PTBP1 axis in cell proliferation, invasion, and metastasis of BUC, we conducted MTT, clone formation, transwell, and wound‐healing assays, respectively. The results indicated that overexpression of *MAFG‐A1* dramatically increased cell viability (Figures 6A and 6B), invasion, and metastasis of BUC cells (Figures 6C and 6D). Additionally, an epithelial marker E‐cadherin protein level was markedly decreased, while the level of mesenchymal marker, vimentin, was significantly increased when *MAFG‐AS1* was overexpressed in T24 and RT4 cells (Figure 6E). By contrast, ectopic overexpression of *MAFG‐AS1* and knockdown of HuR or PTBP1 partly inhibited the promoting effects on proliferation, invasion, metastasis, and EMT of BUC cells induced by *MAFG‐AS1*. Subsequently, we tested the role of *MAFG‐AS1* on BUC in vivo experiments. After knocking down *MAFG‐AS1*, the tumor‐forming ability of the cells was decreased (Figure 7A), the EMT process was inhibited (Figure 7B); in sharp contrast, overexpression of *MAFG‐AS1* exerted a significant promoting effect on tumor growth and EMT process (Figures 7A and 7B). In addition, it was found that the tumorigenic ability of *MAFG‐AS1* could be weakened by knockdown of HuR or PTBP1. Consistently, EMT process was inhibited with cotransfection of *MAFG‐AS1* and knockdown of HuR or PTBP1 compared with overexpression of *MAFG‐AS1* (Figures 7A and 7B). IHC to detect tumor proliferation‐related indicators Ki67 also confirmed this result (Figure 7C). Therefore, the findings of this study uncover a novel molecular mechanism in the progression of BUC via *MAFG‐AS1*/HuR/PTBP1 axis, which promotes cell proliferation, invasion, and EMT in BUC.

## 4 DISCUSSION

Emerging and accumulating studies reveal that a number of lncRNAs can regulate the genesis and development of tumors through a variety of mechanisms. *MAFG‐AS1* is a newly discovered oncogenic lncRNA in several cancers.^37^, ^38^ However, the role of *MAFG‐AS1* in BUC remains poorly understood. Here, we found that *MAFG‐AS1* expression is upregulated in BUC tissues based on TCGA and GEO (GSE31189) databases. Then, in vitro and in vivo experiments revealed that *MAFG‐AS1* could promote cell proliferation, invasion, metastasis, and EMT progression in BUC. Further analysis found that increased *MAFG‐AS1* in BUC patients was correlated with poor prognosis and advanced TNM stage. Therefore, *MAFG‐AS1* may be a prognostic biomarker for BUC.

HuR, an RBP, belongs to the human/embryo lethal abnormal vision (Hu/ELAV) RBP family, and regulates mRNA stability, translation, and miRNA production,^39^ and its activity is mainly regulated by nuclear translocation,^40^ phosphorylation,^41^ and ubiquitination.^42^ Recently, studies have shown that lncRNAs participate in regulation of HuR function. lincRNA*‐UFC1* ^19^ can directly bind to HuR protein and stabilize its expression, while lncRNA *OCC‐1* can promote its ubiquitination at the posttranslational level to degrade the protein level of HuR.^43^ In this study, it was predicted that *MAFG‐AS1* could bind to HuR through HDOCK, and verified by PRM and RIP analysis. Importantly, *MAFG‐AS1* was positively correlated with HuR at the transcriptional level according to ChIPBase 2.0 and starBase 2.0. Furthermore, overexpression of *MAFG‐AS1* remarkably enhanced HuR protein levels, indicating that *MAFG‐AS1* is highly likely to stabilize the expression of HuR protein upon binding. Ubiquitination is involved in the stability of proteins and eventually leads to their specific degradation. Therefore, we identified several deubiquitinating enzymes via several online databases, such as UALCAN, ChIPBase 2.0, and GEPIA. We identified that USP5 was positively correlated with HuR and *MAFG‐AS1* as well with poor prognosis in BUC. USP5 is a deubiquitinating enzyme that has been reported to facilitate tumorigenesis in multiple cancers.^44^, ^45^ Furthermore, we predicted, using HDOCK and catRAPID, that there are binding sites between USP5, HuR, and *MAFG‐AS1*. This analysis suggested that *MAFG‐AS1* is likely to promote tumorigenesis and progression of BUC by inhibiting the ubiquitination of HuR via recruiting USP5. It was further validated that USP5 directly binds to HuR by Co‐IP assays. We observed that overexpression of *MAFG‐AS1* could upregulate HuR protein level in T24 and RT4 cells, which could be partly inhibited by silencing USP5. These data proved that *MAFG‐AS1* could directly bind to HuR and stabilize it by recruiting the USP5 deubiquitinating enzyme in BUC. Our results provided a reasonable answer to the question of why HuR is overexpressed in BUC raised in previous studies. However, further investigation is needed to identify whether HuR is an independent prognostic factor.

HuR could confer transcriptional stability and promote translation by binding to ARE of the 3′ untranslated region of mRNA in cells.^46^, ^47^ As a result, we hypothesized that HuR might play a role in BUC by regulating certain key mRNAs. PTBP1, a known hnRNPI, could promote the progression of multiple tumors.^48^, ^49^ Moreover, it was found that highly expressed PTBP1 facilitates genesis and development of tumors, and was associated with poor prognosis as well as lymph node metastasis in BUC.^50^, ^51^ As yet, no studies have explored the molecular mechanism involved in the high expression of PTBP1 in BUC. Interestingly, we found that HuR is positively correlated with PTBP1 based on the ChIPBase and GEO database, which is consistent with previous results.^52^ Stability tests with RIP and RNA revealed that HuR promotes the translational level of PTBP1 by binding and stabilizing its mRNA. Furthermore, function recovery assays showed that overexpression of *MAFG‐AS1* and knockdown of HuR or PTBP1 reduced cell proliferation, metastasis, and inhibited EMT process in BUC. We confirmed that *MAFG‐AS1* facilitates carcinogenesis and progression in BUC by upregulating PTBP1 via directly binding and stabilizing HuR. Our results also suggest that PTBP1 serves as an oncogene in BUC, and clarified the possible molecular mechanism of overexpressed PTBP1.

In conclusion, we confirmed that *MAFG‐AS1* is markedly upregulated in BUC tissues, and higher expressed *MAFG‐AS1* is associated with progression of BUC and poor survival of BUC patients. On the molecular level, *MAFG‐AS1* could promote cell proliferation, metastasis, and EMT process via the *MAFG‐AS1*/HuR/PTBP1 axis in BUC. We reveal that *MAFG‐AS1* may be a novel target and a potential biomarker for BUC treatment. Targeting *MAFG‐AS1*/HuR/PTBP1 axis can provide new therapeutic avenues for clinical treatment of BUC.

## CONFLICT OF INTEREST

The authors declare that there is no conflict of interest.

## AUTHOR CONTRIBUTIONS

Mengqing Xiao and Ke Cao designed the study, analyzed and interpreted the data, and wrote the manuscript. Jianye Liu, Liang Xiang, Kai Zhao, Dong He, Qinghai Zeng, Yuxing Zhu, and Yeyu Zhang contributed to data acquisition, analysis, and interpretation. Jianye Liu and Qun Zhang provided clinical database compilation and analysis. Hao Bo and Xingyu Chen performed all the bioinformatics analysis. Yan Liu, Xiaoming Liu, Lian Gong, Ying Bao, Yi Hu, Yaxin Cheng, Liping Deng, and Rongrong Zhu carried out the experiments. Dan Xie, Minhua Deng, Xiaowei Xing, Ming Zhou, Wei Xiong, Yanhong Zhou, Jianda Zhou, and Xiaohui Li provided technical expertise and support. All the authors have seen and approved the final version of the manuscript, and agreed to submit the manuscript for consideration of publication in this journal.

## ETHICS STATEMENT

This study was approved by the Third Xiangya Hospital of Central South University, and informed consent was obtained from all the patients. All the experiments in this study were conducted following the corresponding regulations and guidelines.

## Supporting information

Supplementary Figure S1 *MAFG‐AS1* is highly expressed and negatively correlated with prognosis in BUC. A, GEPIA was used to analyze the expression of *MAFG‐AS1* in most types of cancer. B, High *MAFG‐AS1* expression was associated with advanced clinical stages using GEPIA database. C, Expression of *MAFG‐AS1* was identified by RT‐qPCR in cancer and normal tissues of bladder. D, Patients with high *MAFG‐AS1* expression had shorter DFS in BUCs; data from GEPIA. Bars represent standard deviation, ns *P* > .05, *P < .05, **P < .01, ***P < .001

Supplementary Figure S2 Potential deubiquitinating enzymes that may be recruited by *MAFG‐AS1*. A‐E, Five deubiquitinating enzymes such as UCHL5 (A), USP5 (B), COPS6 (C), PSMD14 (D), and OTUB1 (E) were selected using UALCAN and ChIPBase 2.0 database. F‐J, The transfection efficiency of si‐UCHL5 (F), si‐USP5 (G), si‐COPS6 (H), si‐PSMD14 (I), and si‐OTUB1 (J) was shown by Western blot in T24 and RT4 cells

Supplementary Figure S3 Correlation between *MAFG‐AS1*, USP5, and HuR was predicted by CHIPBASE2.0 and GEO datasets in BUC. A, The correlation between *MAFG‐AS1* and HuR in BUC was predicted by ChIPBase 2.0. B‐F, The correlation between HuR and PTBP1 in BUC was predicted by ChIPBase 2.0 (B), GSE83586 (C), GSE13507 (D), GSE31684 (E), and GSE124305 (F). G, The correlation between HuR and USP5 was predicted by ChIPBase 2.0 in BUC. H and I, The correlation between *MAFG‐AS1* and USP5 was predicted by GSE124305 (H) and GSE87304 (I) in BUC.

Supplementary Figure S4 Bioinformatics analysis based on catRAPID and HDOCK databases. A, The potential binding sites between *MAFG‐AS1* and HuR were detected by catRAPID databases. B and C, The potential binding sites between *MAFG‐AS1* and USP5 were detected using catRAPID (B) and HDOCK (C) databases. D, The potential site between HuR and USP5 was detected by HDOCK database.

TableS1

TableS2

TableS3

TableS4

TableS5

TableS6

TableS7

## Data Availability

All the data generated or analyzed during this study are included in this published article. The datasets supporting the conclusions of this article are included within the article and its additional supporting files.
