# Supplementary figures and images for: MAFG‐AS1 promotes tumor progression via regulation of the HuR/PTBP1 axis in bladder urothelial carcinoma
Source: Clin Transl Med. 2020 Dec 16;10(8):e241. doi: 10.1002/ctm2.241 (PMC7744027; doi:10.1002/ctm2.241)

# A

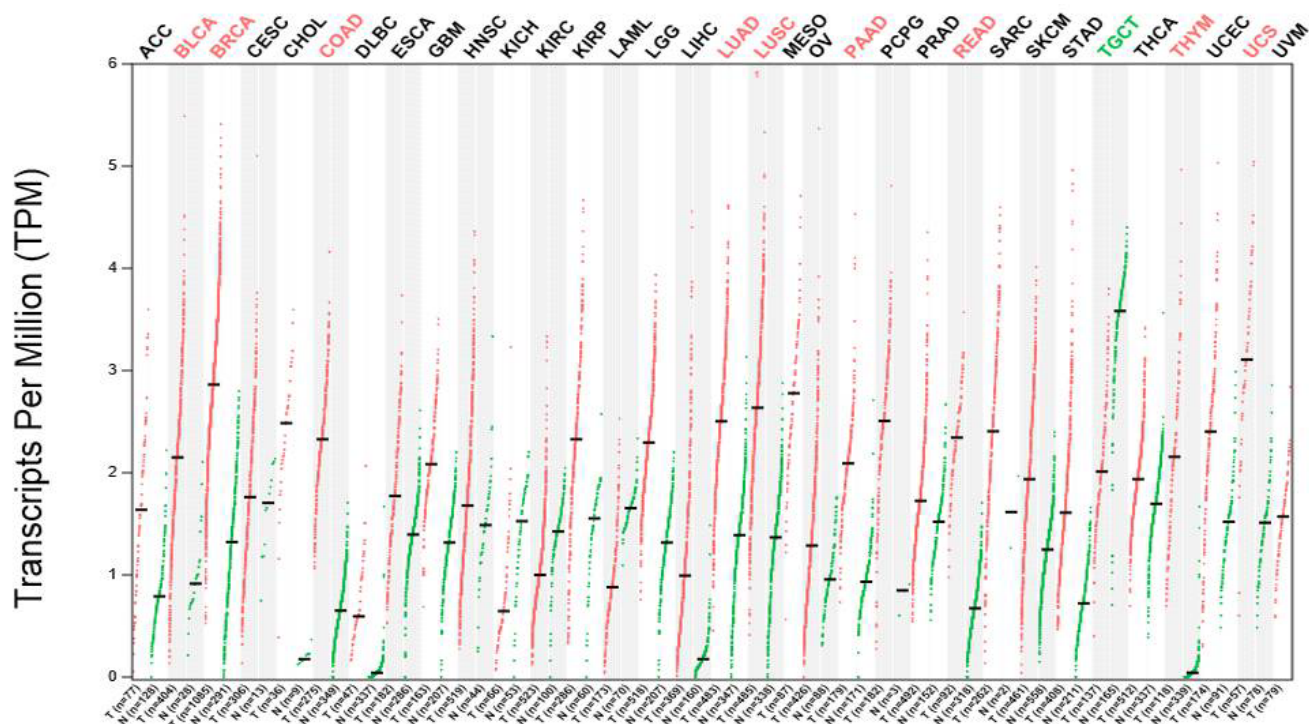

# B

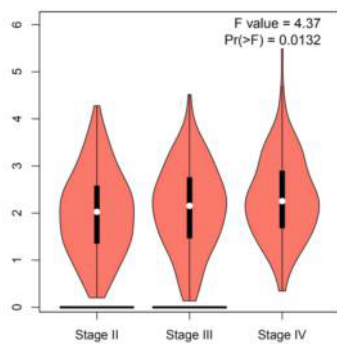

**C**

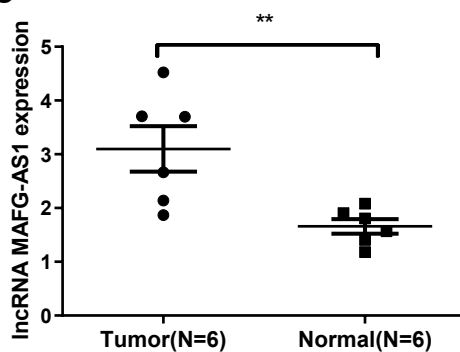

D

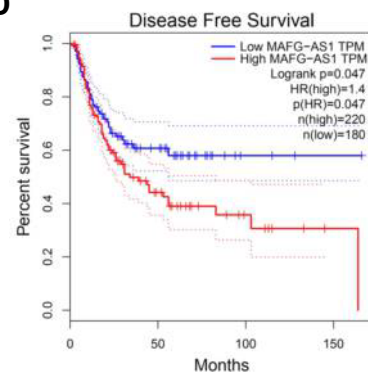

Supplement: Supplementary file 1 — Supplementary Figure S1 MAFG‐AS1 is highly expressed and negatively correlated with prognosis in BUC. A, GEPIA was used to analyze the expression of MAFG‐AS1 in most types of cancer. B, High MAFG‐AS1 expression was associated with advanced clinical stages using GEPIA database. C, Expression of MAFG‐AS1 was identified by RT‐qPCR in cancer and normal tissues of bladder. D, Patients with high MAFG‐AS1 expression had shorter DFS in BUCs; data from GEPIA. Bars represent standard deviation, ns P > .05, *P < .05, **P < .01, ***P < .001 [file CTM2-10-e241-s001.pdf]

**A**

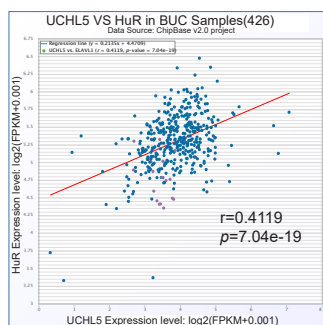

**B**

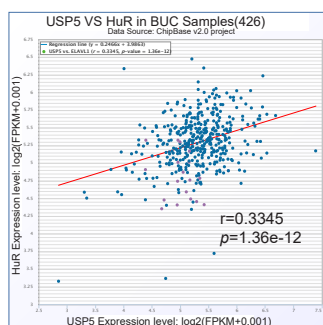

**C**

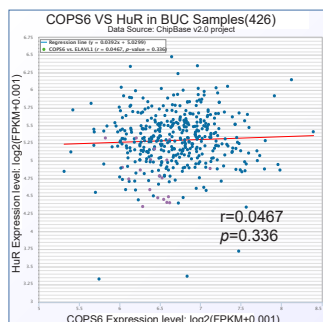

**D**

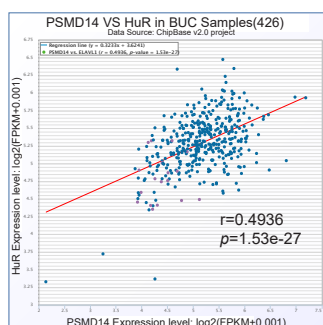

**E**

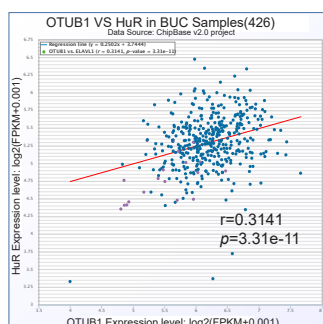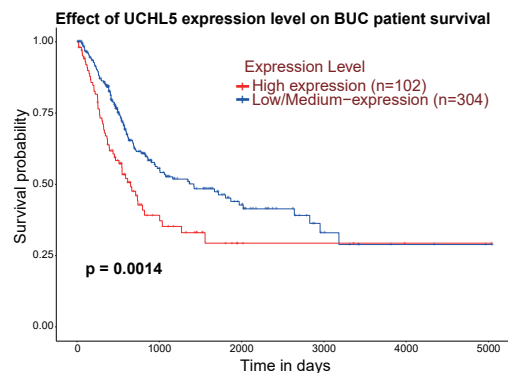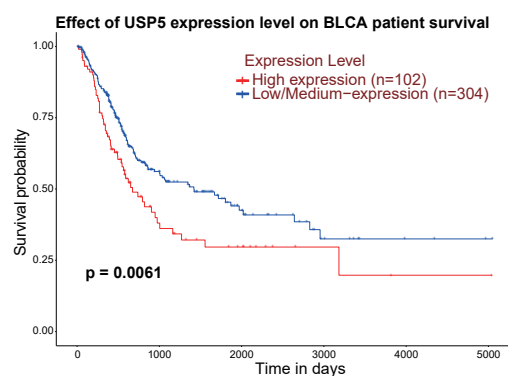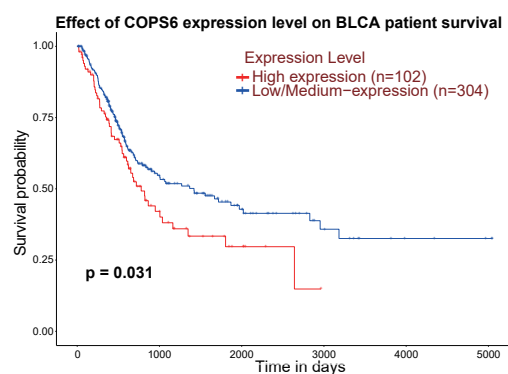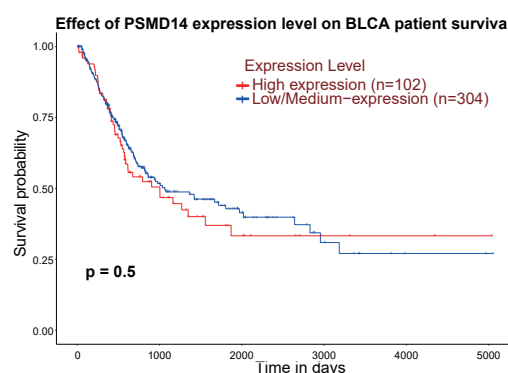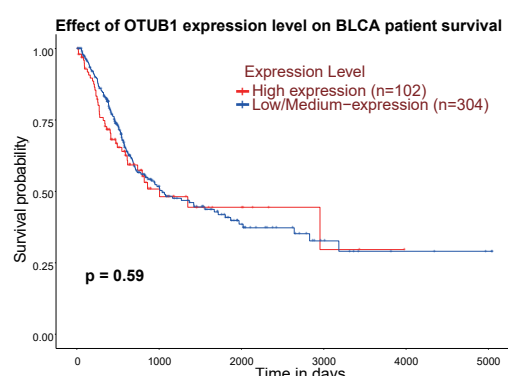

**F**

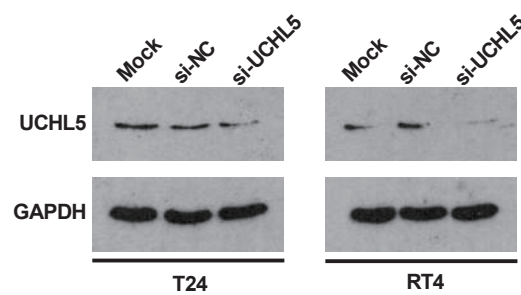

**G**

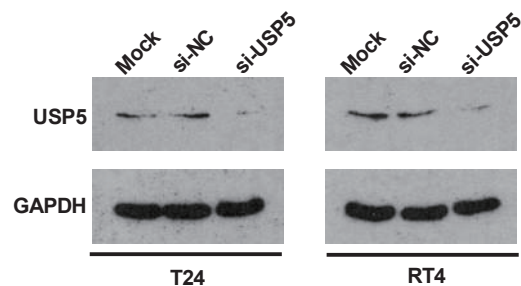

**H**

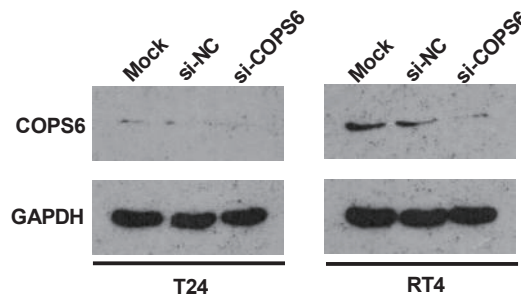

**I**

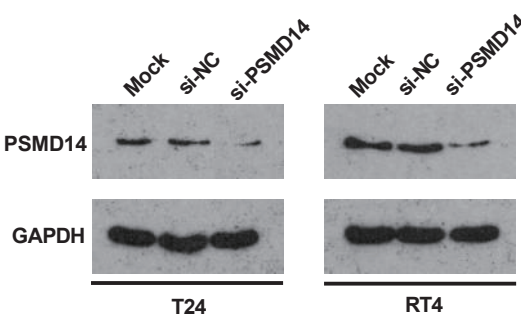

**J**

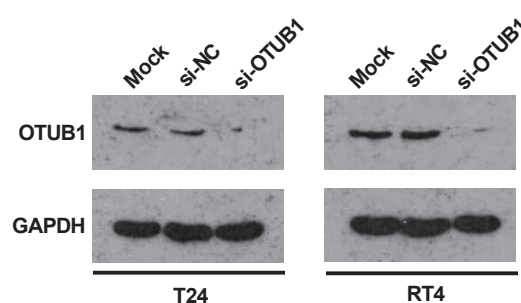

Supplement: Supplementary file 2 — Supplementary Figure S2 Potential deubiquitinating enzymes that may be recruited by MAFG‐AS1. A‐E, Five deubiquitinating enzymes such as UCHL5 (A), USP5 (B), COPS6 (C), PSMD14 (D), and OTUB1 (E) were selected using UALCAN and ChIPBase 2.0 database. F‐J, The transfection efficiency of si‐UCHL5 (F), si‐USP5 (G), si‐COPS6 (H), si‐PSMD14 (I), and si‐OTUB1 (J) was shown by Western blot in T24 and RT4 cells [file CTM2-10-e241-s002.pdf]

A

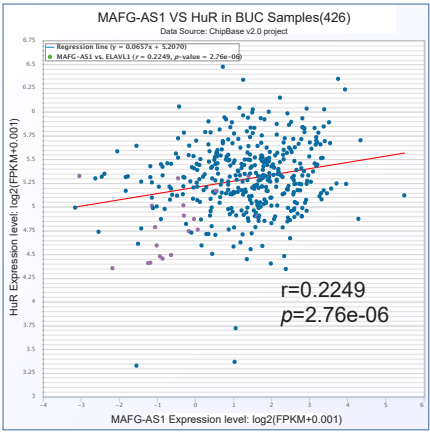

C

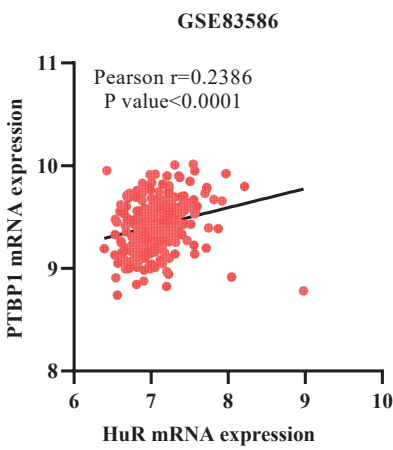

D

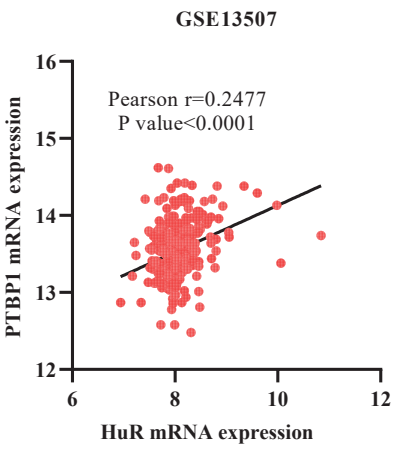

B

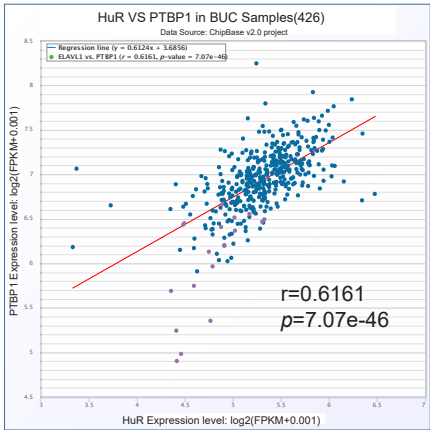

E

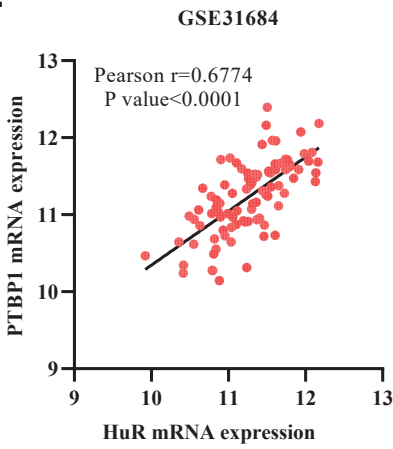

F

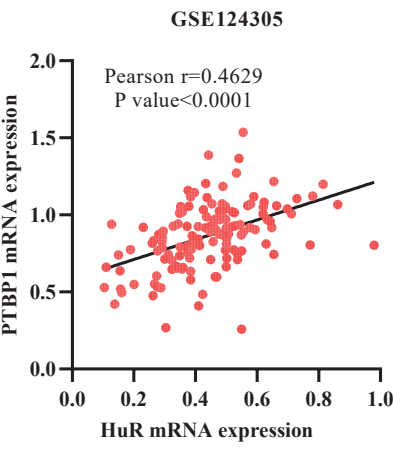

G

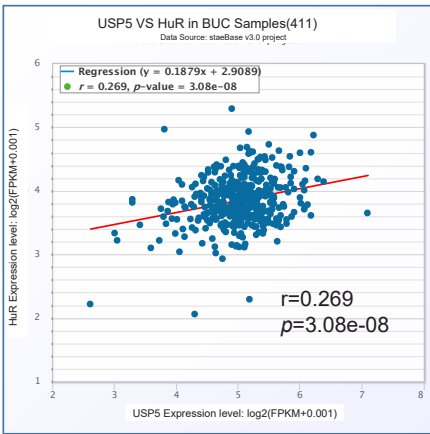

H

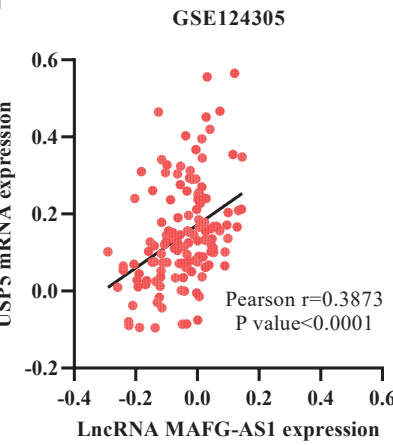

I

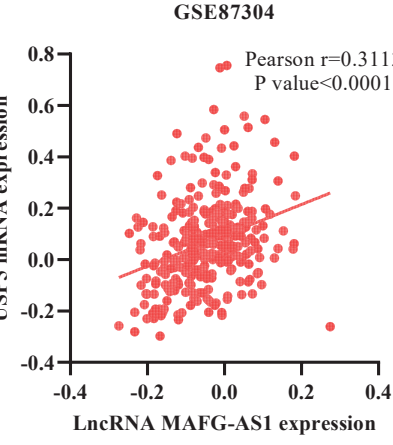

Supplement: Supplementary file 3 — Supplementary Figure S3 Correlation between MAFG‐AS1, USP5, and HuR was predicted by CHIPBASE2.0 and GEO datasets in BUC. A, The correlation between MAFG‐AS1 and HuR in BUC was predicted by ChIPBase 2.0. B‐F, The correlation between HuR and PTBP1 in BUC was predicted by ChIPBase 2.0 (B), GSE83586 (C), GSE13507 (D), GSE31684 (E), and GSE124305 (F). G, The correlation between HuR and USP5 was predicted by ChIPBase 2.0 in BUC. H and I, The correlation between MAFG‐AS1 and USP5 was predicted by GSE124305 (H) and GSE87304 (I) in BUC. [file CTM2-10-e241-s003.pdf]

Supplementary Figure 4

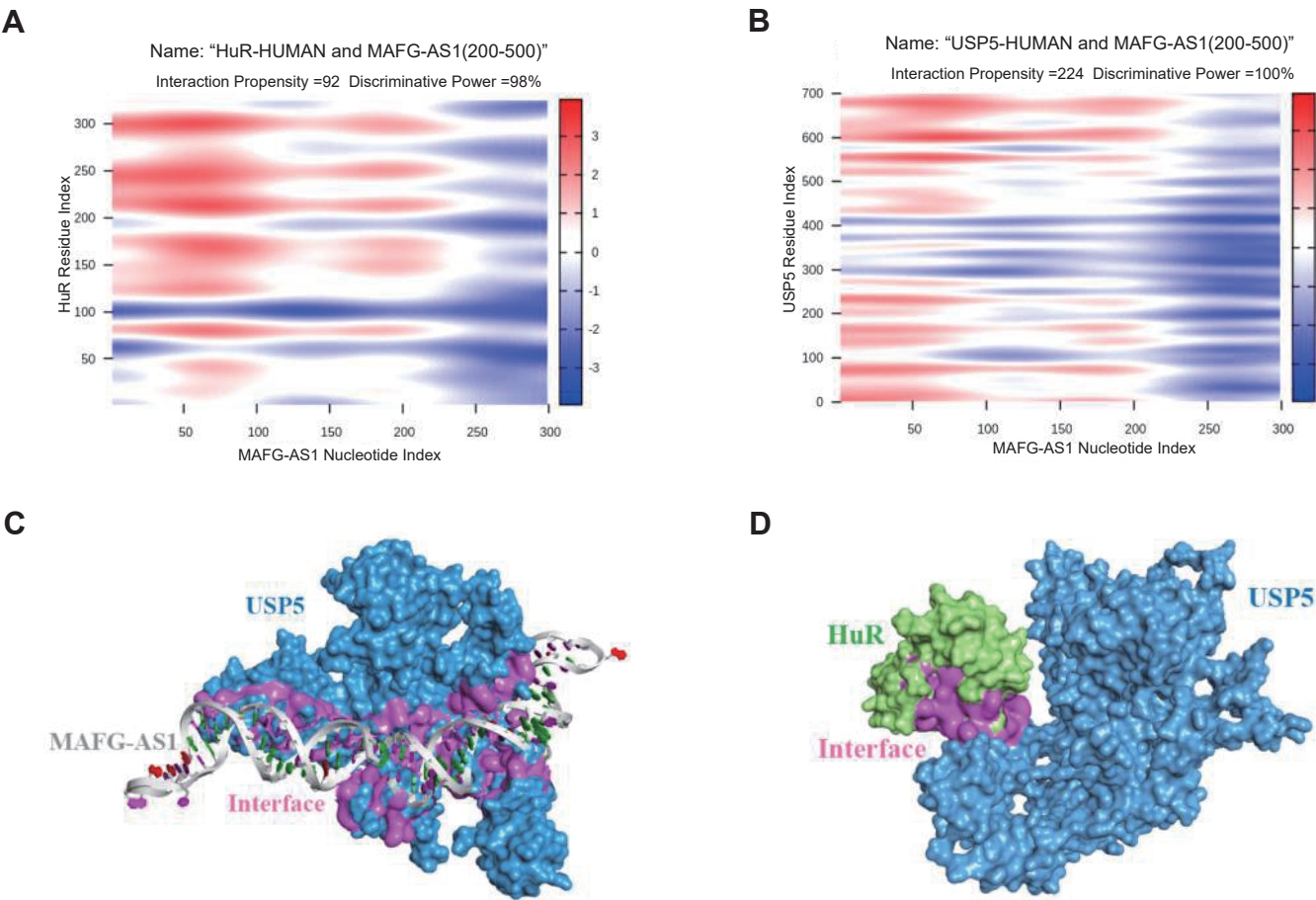

Supplement: Supplementary file 4 — Supplementary Figure S4 Bioinformatics analysis based on catRAPID and HDOCK databases. A, The potential binding sites between MAFG‐AS1 and HuR were detected by catRAPID databases. B and C, The potential binding sites between MAFG‐AS1 and USP5 were detected using catRAPID (B) and HDOCK (C) databases. D, The potential site between HuR and USP5 was detected by HDOCK database. [file CTM2-10-e241-s004.pdf]
