# Supplementary material for: MAFG‐AS1 promotes tumor progression via regulation of the HuR/PTBP1 axis in bladder urothelial carcinoma
Source: Clin Transl Med. 2020 Dec 16;10(8):e241. doi: 10.1002/ctm2.241 (PMC7744027; doi:10.1002/ctm2.241)
Supplement: Supplementary file 5 — TableS1 [file CTM2-10-e241-s005.pdf]

Table S1: Correlation of MAFG-AS1 expression in tissue with patients' clinicopathological variables in 102 cases of BUC.

		MAFG-AS1 expression		
Variables	Cases	Low(%)	High(%)	*P*- value[a]
Age (years)				0.541
≤65[b]	53	27(50.9)	26(49.1)	
＞65	49	22(44.9)	27(55.1)	
Sex				0.657
Male	87	41(47.1)	46(52.9)	
Female	15	8(53.3)	7(46.7)	
Tumor size (cm)				0.16
≤3.4[c]	53	29(54.7)	24(45.3)	
＞3.4	49	20(40.8)	29(59.2)	
Tumor multiplicity				0.086
Unifocal	22	7(31.8)	15(68.2)	
Multifocal	80	42(52.5)	38(47.5)	
Tumor grade				0.616
G1	15	7(46.7)	8(53.3)	
G2	45	24(53.3)	21(46.7)	
G3	42	18(42.9)	24(57.1)	
pT category				**0.009**
pTa/pT1	28	18(64.3)	10(35.7)	
pT2	40	18(45.0)	22(55.0)	
pT3	25	13(52.0)	12(48.0)	
pT4	9	0(0.0)	9(100.0)	
pN category				**0.002**
pN-	80	45(56.3)	35(43.8)	
pN+	22	4(18.2)	18(81.8)	

[a]Chi-square test; [b]median age; [c]median size; BUC: bladder urothelial carcinom.
