# Supplementary material for: MAFG‐AS1 promotes tumor progression via regulation of the HuR/PTBP1 axis in bladder urothelial carcinoma
Source: Clin Transl Med. 2020 Dec 16;10(8):e241. doi: 10.1002/ctm2.241 (PMC7744027; doi:10.1002/ctm2.241)
Supplement: Supplementary file 6 — TableS2 [file CTM2-10-e241-s006.pdf]

Table S2: Univariate analysis of factors associated with survival of 102 patients

Variable	HR (95% CI)	*P*- value
MAFG-AS (high *vs.* low)	7.336 (2.851-18.878)	**＜0.001**
Age (＞65 *vs.* ⩽65)	1.051 (0.546-2.025)	0.881
Gender (female *vs.* male)	1.123 (0.466-2.707)	0.797
Tumor size (＞3.4 *vs.* ⩽3.4)	1.179 (0.618-2.251)	0.617
Tumor multiplicity (multifocal *vs.* unifocal)	1.079 (0.520-2.236)	0.839
Tumor grade (G3 *vs.* G2 *vs.* G1)	1.264 (0.784-2.039)	0.336
pT category (pT4 *vs.* pT3 *vs.* pT2 *vs.* pTa/pT1	2.462 (1.687-3.592)	**＜0.001**
pN category (pN+ *vs.* pN-)	13.273 (6.060-29.072)	**＜0.001**

HR: hazard ratio; CI: confidence interval; BUC: bladder urothelial carcinom.
