# Supplementary material for: MAFG‐AS1 promotes tumor progression via regulation of the HuR/PTBP1 axis in bladder urothelial carcinoma
Source: Clin Transl Med. 2020 Dec 16;10(8):e241. doi: 10.1002/ctm2.241 (PMC7744027; doi:10.1002/ctm2.241)
Supplement: Supplementary file 7 — TableS3 [file CTM2-10-e241-s007.pdf]

Table S3: Multivariate analysis of prognostic factors on survival (Cox regressic

Variable	HR (95% CI)	*P* -value
MAFG-AS1 (high *vs.* low)	4.603 (1.708-12.407)	**0.003**
pT category (pT4 *vs.* pT3 *vs.* pT2 *vs.* pTa/pT	1.236(0.783-1.951)	0.364
pN category (pN+ *vs.* pN-)	6.044(2.124-17.201)	**0.001**

HR: hazard ratio; CI: confidence interval.
