# Supplementary material for: MAFG‐AS1 promotes tumor progression via regulation of the HuR/PTBP1 axis in bladder urothelial carcinoma
Source: Clin Transl Med. 2020 Dec 16;10(8):e241. doi: 10.1002/ctm2.241 (PMC7744027; doi:10.1002/ctm2.241)
Supplement: Supplementary file 11 — TableS7 [file CTM2-10-e241-s011.docx]

**Table S7. Public databases used in this study**

| **Public databases** | **websites** |
| --- | --- |
| Cancer RNA-Seq Nexus | [http://syslab4.nchu.edu.tw/index.jsp](https://mc.manuscriptcentral.com/ctm2?DOWNLOAD=TRUE&PARAMS=xik_2xQobB2gdaMfkq7Rq7qLFdCXJVo3VJc7ZZkEZuNGg7DwiPVN1EG1FtTtLWXqzyQDoUTqPovK7V7sJP3UtFBHFXpUZAr1nkkFHPM3skbjqNRM5VANkqbQC9KUgMYxB3qv4UujXfprRW5PBALdntimDS8cSJQXbyjeJ78TcjvQqjzDUmqSnGtovKYq5jR6AYJGDaxgc1QaeviWhJhQyZpVRfCQdFYW3gpS5pmXYxEvdcPGyCysfGkC4UY7FRU1NVin2nHJvt1) |
| TANRIC | http://bioinformatics.mdanderson.org/main/TANRIC:Overview |
| XENA | <https://xena.ucsc.edu/> |
| GEPIA | <http://gepia.cancer-pku.cn/> |
| UALCAN | <http://ualcan.path.uab.edu/> |
| CHIPBASE | http://rna.sysu.edu.cn/chipbase/ |
| catRAPID | http://service.tartaglialab.com/page/catrapid_group |
| STARBASE | <http://starbase.sysu.edu.cn/> |
| HDOCK | <http://hdock.phys.hust.edu.cn/> |
| POSTAR | http://lulab.life.tsinghua.edu.cn/postar/index.php |
| Metascape | http://metascape.org/ |
| Molecular Signatures Database | http://software.broadinstitute.org/gsea/msigdb |

**Table S8. Primers used in this study**

| Genes | Forward primer (5‘-3’) | Reverse primer (5‘-3’) |
| --- | --- | --- |
| *MAFG-AS1* | GAAGGTGTTCCGTGGTCAGT | GATGAGTGTGCGGAGTGAGA |
| *HuR* | GGGTGACATCGGGAGAACG | CTGAACAGGCTTCGTAACTCAT |
| *PTBP1* | AGCGCGTGAAGATCCTGTTC | CAGGGGTGAGTTGCCGTAG |
| *GAPDH* | GGAGCGAGATCCCTCCAAAAT | GGCTGTTGTCATACTTCTCATGG |
| *U1* | GGGAGATACCATGATCACGAAGGT | CCACAAATTATGCAGTCGAGTTTCCC |
|  |  |  |
